# Polarity‐Controlled Volatile HfO_2_ Memristors with Bimodal Conductance for Neuromorphic Synapses and Reservoir Computing

**DOI:** 10.1002/advs.202515926

**Published:** 2025-11-03

**Authors:** Yuseong Jang, Chanmin Hwang, Myoungsu Chae, Taegi Kim, Hee‐Dong Kim

**Affiliations:** ^1^ Department of Semiconductor Systems Engineering Department of Electrical Engineering and Convergence Engineering for Intelligent Drone Sejong University 209, Neungdong‐ro Gwangjin‐gu Seoul 05006 South Korea; ^2^ Institute of Industrial Science Department of Electrical Engineering The University of Tokyo 4‐6‐1 Komaba Meguro Tokyo 153‐8505 Japan

**Keywords:** bimodal, field‐induced dipole, ion bomb, RC system, RRAM

## Abstract

In this work, an HfO_2_‐based memristor exhibiting bimodal switching, wherein the device's conductance is modulated not only by the input stimulus but also by the polarity of the read voltage, is introduced. Uniquely, this device demonstrates reliable short‐term memory (STM)‐like behavior and supports 16 well‐separated conductance states through 4‐bit pulsed inputs. Remarkably, under the same input conditions, reversing the polarity of the read voltage results in 16 more different conductance states, thereby doubling the number of levels that can be distinguished per cell. Employing the proposed device, a reservoir computing (RC) system, which takes advantage of this rich representational capability, is created. The system achieves a high classification accuracy of 98.81% on the MNIST dataset. These results show how powerful memristor‐based architectures can be and how this device could be a compact and energy‐efficient platform for the next generation of neuromorphic computing.

## Graphical abstract

This study demonstrates a TiN/HfO_2_/ITO memristor exhibiting field‐induced bimodal volatile switching behavior originating from ion bombardment during sputtering. The dipole‐driven, polarity‐dependent states produce multilevel output responses that expand the reservoir's output dimensionality and enhance encoding diversity. Such a mechanism enables efficient real‐time signal processing, offering a compact and energy‐efficient hardware platform for advanced neuromorphic and reservoir computing applications.

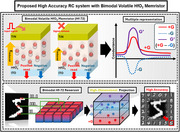

## 1 Introduction

Improvements in semiconductor technology have accelerated the development of intelligent systems such as the Internet of Things, driverless cars, and smart healthcare platforms.^[^ ^1^ ^]^ Due to the exponential growth in data throughput and the need for real‐time processing, there is an urgent need for novel hardware architectures that can handle large volumes of data with high energy efficiency.^[^ ^2^ ^]^ However, the traditional von Neumann architecture, which separates memory and computation units, still makes it difficult to achieve these goals.^[^ ^3^ ^]^ Inspired by the architecture and functioning of biological neural networks, neuromorphic computing has become a viable way to get around this restriction.^[^ ^1^, ^2^, ^3^ ^]^ Neuromorphic systems provide massively parallel processing at a lower power consumption by combining memory and processing in one device.^[^ ^4^, ^5^, ^6^, ^7^ ^]^ To realize such neuromorphic functionality, various device architectures have been investigated across different material platforms. Chang et al. demonstrated an optoelectronic dual‐synapse based on a wafer‐level GaN‐on‐Si device capable of responding to both optical and electrical stimuli, enabling multimodal signal processing.^[^ ^8^ ^]^ Likewise, Li et al. exploited dendritic dislocations in GaN HEMTs to emulate neuromodulation and adaptive learning behaviors.^[^ ^9^ ^]^

Beyond such compound semiconductor–based approaches, memory devices exhibiting analog conductance modulation have also been widely explored for neuromorphic computing hardware. Among the various emerging memory technologies explored for such systems, including ferroelectric random access memory,^[^ ^10^, ^11^ ^]^ spin‐transfer torque magneto resistive random access memory,^[^ ^12^ ^]^ phase‐change memory,^[^ ^13^ ^]^ and resistive switching memory (RRAM),^[^ ^14^, ^15^ ^]^ RRAM stands out due to its potential to mimic synaptic behavior and enable in‐memory computation for efficient pattern recognition and learning.^[^ ^16^, ^17^, ^18^ ^]^ Its potential stems from its simple structure, material versatility, and analog switching controllability, which have continuously motivated the development of RRAM‐based neuromorphic devices.^[^ ^19^, ^20^ ^]^

**Figure 1:**
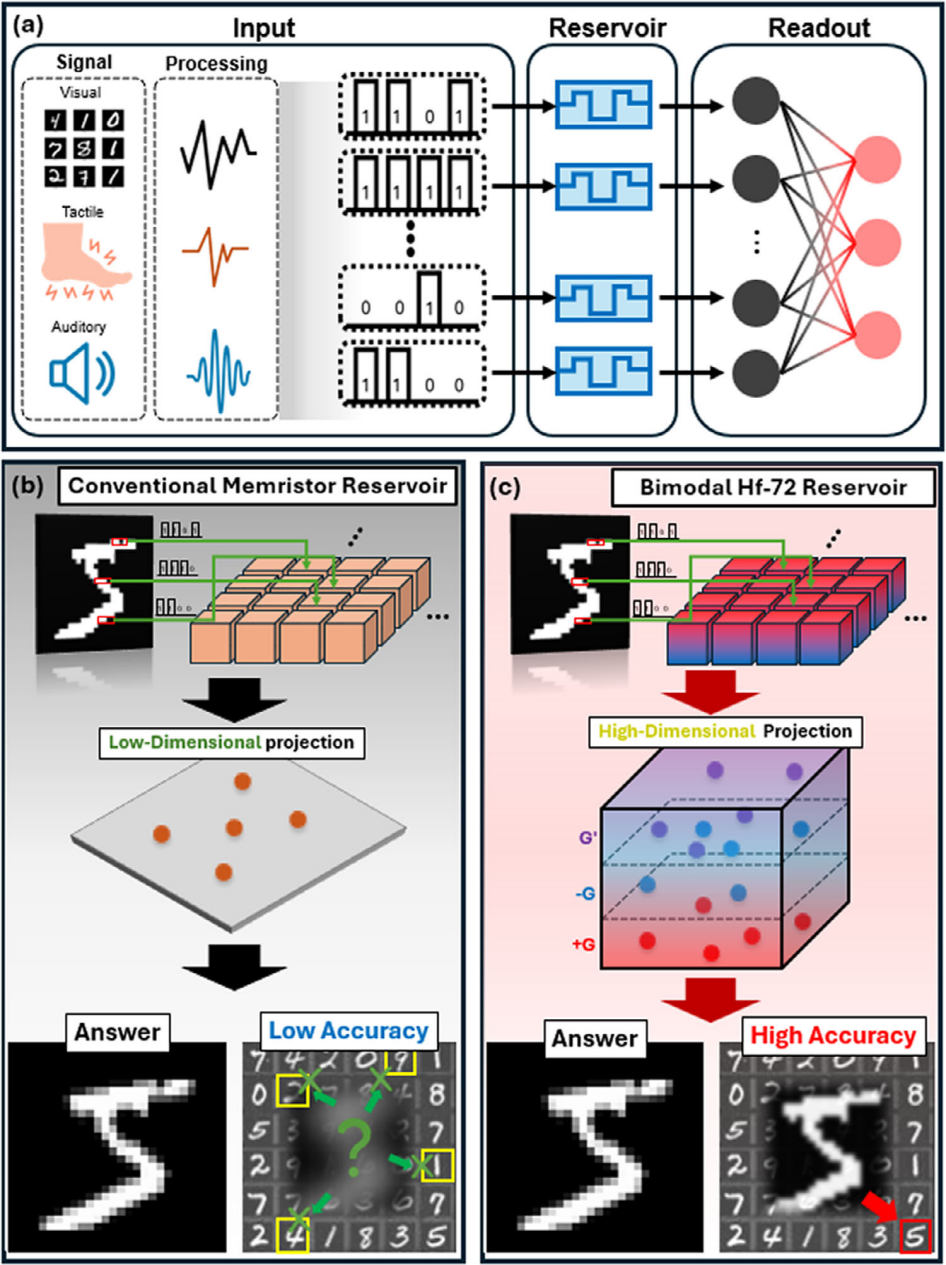
Conceptual illustration of bimodal memristor‐based reservoir computing. a) Schematic representation of a neuromorphic reservoir computing system utilizing a memristor‐based reservoir. b) Conventional memristor reservoir structure and its output response, mapped onto a low‐dimensional state space. c) The proposed bimodal Hf‐72 reservoir, facilitating multichannel encoding and projection into a high‐dimensional output space.

Spiking neural networks (SNNs), which process information through event‐driven spike‐based dynamics, are gaining popularity despite the extensive use of artificial neural networks in neuromorphic systems due to their high energy requirements and restricted temporal modeling capabilities.^[^ ^21^ ^]^ SNN provides conceptual inspiration for reservoir computing (RC), such as especially in regard to its liquid state machine form, by utilizing their sparse activation behavior and temporal dynamics.^[^ ^22^, ^23^ ^]^ In practical implementations, RC models must rely on recent input history, which results in limited utilization of long‐term memory (LTM) capabilities. Consequently, for many temporal tasks that demand fast and adaptive responses, short‐term memory (STM) becomes more critical than long‐term retention. As a result, the recent studies increasingly focus on physical reservoirs that exploit devices with pronounced STM characteristics.^[^ ^5^, ^21^, ^24^ ^]^ RRAM devices with intrinsic temporal dynamics have demonstrated a lot of potential as physical reservoirs, which is illustrated in **Figure** **1**a. They are appealing for hardware implementations of SNN‐based RC systems due to their capacity to encode temporal information as well as consume minimal amounts of energy. The recent research that utilizes RRAM for RC architectures is compiled in **Table** **1**. The majority of devices function via oxygen ion migration or conductive filament (CF) formation. CF‐based devices offer fast switching and high on/off ratios, but they suffer from variability and reliability issues in dense arrays due to filament instability and localized heating.^[^ ^25^ ^]^ Ion migration‐based devices in contrast provide more stable operation^[^ ^26^ ^]^ but with fabrication complexity due to the need for multilayer structures and precise control of oxygen profiles.^[^ ^27^ ^]^ Post‐deposition treatments such as rapid thermal annealing^[^ ^28^ ^]^ and microwave exposure^[^ ^21^ ^]^ have been explored in order to address these limitations, but introduce additional processing steps and costs. Figure S1a (Supporting Information) illustrates that unidirectional conductance modulation is a typical characteristic of conventional RRAM, which hampers its effectiveness in expressing diverse temporal patterns. We address this by introducing a TiN/HfO_2_/ITO RRAM device that exhibits field‐induced dipole‐based bimodal switching (FDS), which is illustrated in Figure S1b (Supporting Information), without the need for post‐processing. Field‐aligned dipole formation is aided by the densification of the HfO_2_ layer caused by ion bombardment during radio‐frequency (RF) sputtering, as shown in Figure S1c (Supporting Information). Bidirectional conductance modulation is produced by these dipoles based on the polarity of the read voltage. Additional details are provided in Note S1 (Supporting Information). Unlike traditional memristors, which generate a single state under fixed read polarity, this bimodal behavior allows for richer output states (+G, –G, G′) from identical inputs, as shown in Figure 1b.

**Table 1:** Summarizing the recent studies on RRAM devices for an RC system.

Structure	Switching type	Operating current [µA]	Operating bias [V]	Generated input channel [#]	MNIST pattern recognition accuracy [%]	Refs.
Au/PZT/SFO/SRO/STO	Ion migration	<100	−4–4	1	90.12	[64]
ITO/ZrO_x_/TaN/SiO2/Si	Ion migration	<100	−2–5	1	91.54	[65]
Ag/FeO_x_/FeWO_x_/Pt	Filament based	<10	−0.4–0.6	1	91.69	[3]
Ru/NiO/NbO_x_/TiO_2_/TiN	Ion migration	<100	−2–2	1	93.0	[66]
TiN/WO_x_/Pt	Ion migration	<10 000	−3–3	1	92	[67]
Au/SiO_2_/CoO_x_/ITO	Ion migration	<30	−3–5	1	93.39	[68]
Pt/TaO_x_/TiN	Ion migration	<1000	−1.5–2	1	92.35	[5]
Pt/Ag/ZrO_2_/TiN	Ion migration	<1	0–1.5	1	87.1	[69]
Ag/CH‐P/PMMA//NiO/FTO	Filament based	<15 000	−2–2	1	94.1	[70]
ITO/HfTiO_x_/Au NP/TiSiO_x_/TaN	Filament based	<700	−1.35–1.5	1	82.36	[15]
ITO/ZnO/IGZO/ZnO/ITO	Filament based	<100	−2–2	1	87.79	[18]
ITO/Al_2_O_3_/Nb:STO/Ti	Ion migration	<100	−3–3	1	91.6	[71]
Pd/Au/WO_x_/W/SiO_2_/Si	Ion migration	<175	−1.5–1.5	1	88.1	[60]
ITO/p‐NiO/n‐IGZO/Ti/Pt	optoelectronic	<0.00003	−0.2–0.2	1	95.2	[72]
Cu/NC h‐BN/Cr/Au	Filament based	<1	0–0.7	1	93.6	[73]
TiN/HfO_2_/ITO	Dipolar	<10	−5–5	3	98.81	This work

Despite the fact that this type of FDS has been seen in memristive devices before, its use has been restricted to basic memory applications.^[^ ^29^, ^30^, ^31^ ^]^ On the other hand, our method makes use of this behavior to achieve three different conductance states from a single pulse input, which allows for the device‐level embedding of multichannel information. Without the need for extra circuitry or computational conversion, this physical increase in output dimensionality immediately improves the reservoir's capacity for temporal encoding. The device is also made with a CMOS‐compatible RF sputtering process and has strong cycle‐to‐cycle and device‐to‐device reproducibility, which demonstrates its usefulness for scalable neuromorphic systems. By increasing the reservoir's output dimensionality, as illustrated in Figure 1c, our device improves the classification accuracy in time‐dependent tasks and increases the diversity of input encoding.^[^ ^32^ ^]^ This behavior is therefore similar to spike‐based information processing that is seen in SNN, which is where identical input pulses produce different output states depending on the intrinsic device state and read conditions. Together with conductance relaxation and paired‐pulse facilitation, these features show that our device supports its applicability in neuromorphic RC frameworks by embodying significant SNN‐inspired temporal dynamics at the hardware level.^[^ ^33^, ^34^ ^]^

## 2 Results and Discussion

The RF power was fixed at 100 W in order to ensure that the ion bombardment effect during RF sputtering consistently induced the accumulation of surface energy on the substrate. The deposition duration was varied at 5, 12, and 20 min in order to examine its influence on the device states. To quantitatively compare the energy delivered to the device, the energy density per unit area was defined as shown in Equation (1), where *A* represents the substrate area (1.5 × 1.5 cm^2^).

(1) $$\varepsilon =\frac{E_{\mathrm{total}}}{A}$$

The calculated energy densities for each sample were 1.33 × 10⁴, 3.2 × 10⁴, and 5.33 × 10⁴ J cm^−^^2^, which indicate a linear accumulation of energy with an increasing deposition time. The cross‐sectional structures of the corresponding devices were examined using field‐emission scanning electron microscopy (FE‐SEM), as shown in Figure S2 (Supporting Information). The HfO_2_ thicknesses were confirmed to be 18 nm (Hf‐18), 45 nm (Hf‐45), and 72 nm (Hf‐72), respectively, in accordance with the deposition durations.

**Figure 2:**
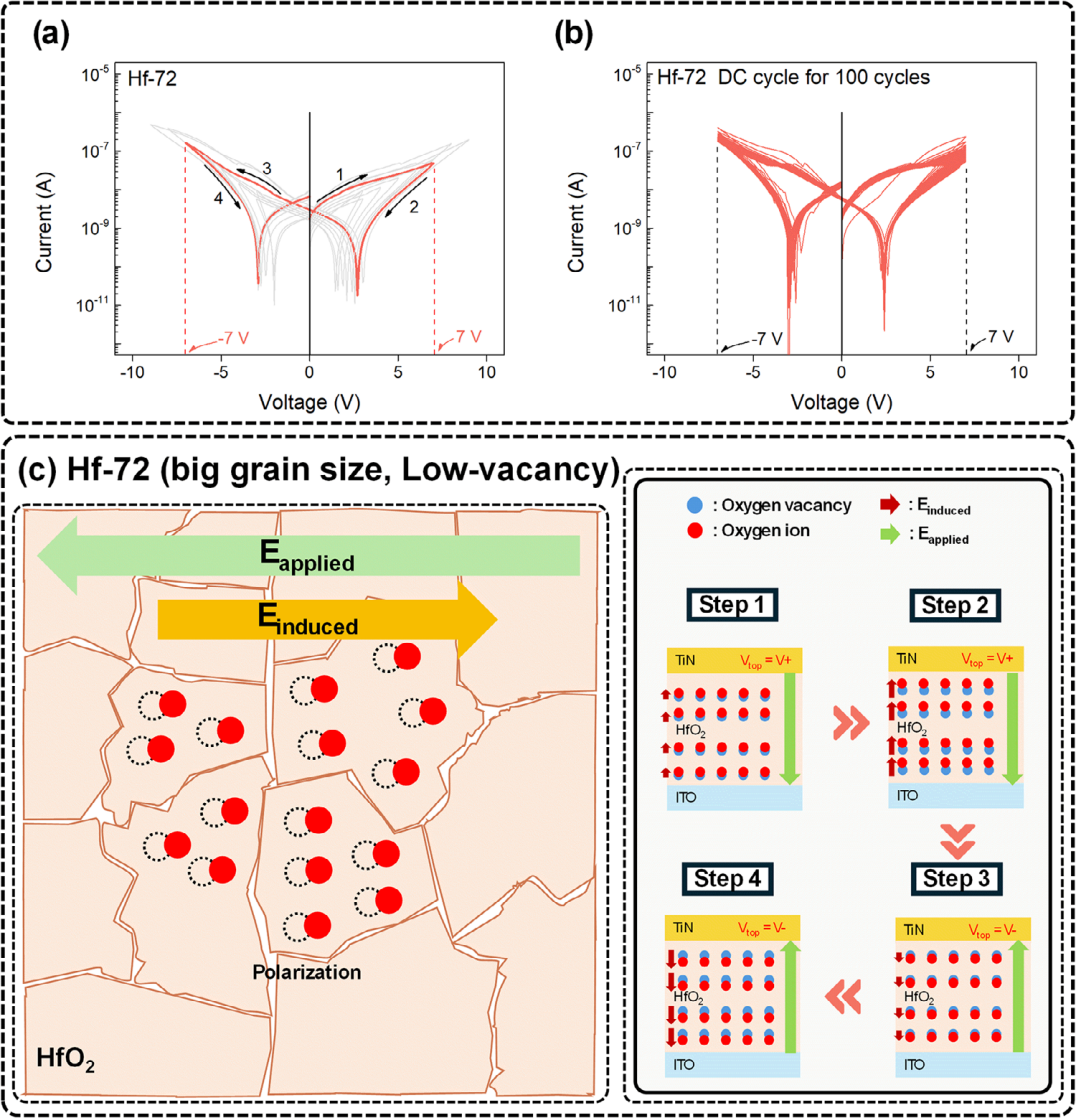
Resistive switching and switching mechanism of the Hf‐72 device. a) *I–V* characteristics of the Hf‐72 device. b) Hundred consecutive DC switching cycles of a single Hf‐72 device under a voltage sweep from –7 to +7 V. c) Schematic representation of the field‐induced dipole‐based switching mechanism in the Hf‐72 device.

The current‐voltage (*I–V*) characteristics of Hf‐18 and Hf‐45 are shown in Figure S3a,b (Supporting Information). The Hf‐18 device exhibits typical filament‐based RRAM behavior. Additional electrical characteristics for Hf‐18 are provided in Figure S3c,d(Supporting Information). In contrast, Figure S3b (Supporting Information) shows that the Hf‐45 device initiates partial FDS, but with relatively low operational stability. Instability in the *I–V* curve is particularly pronounced in the high‐voltage regime, where abrupt switching events frequently occur during FDS. A more detailed discussion is available in Note S3 (Supporting Information). In contrast, the *I–V* characteristics of the Hf‐72 device, shown in **Figure** **2**a, reveal behavior distinct from that of the other samples. Under the highest accumulated energy condition, Hf‐72 does not exhibit typical filament‐based switching. Instead, it displays abnormal bipolar resistive switching (ABRS) and a gradual resistance change. These behaviors imply that the migration of oxygen ions and the subsequent development of a field‐induced dipole within the HfO_2_ layer constitute the predominant switching mechanism.^[^ ^35^ ^]^

Under voltage sweeps, the Hf‐72 device shows a stable transition from a low‐resistance state (LRS) to a high‐resistance state (HRS), and when the external bias is removed, it returns to the LRS on its own. Notably, in contrast to the general observed ABRS with negligible current at zero field,^[^ ^36^, ^37^, ^38^ ^]^ a significant residual current remains at 0 V, and symmetric current minima are observed around ±2–3 V. This resulted in a butterfly‐shaped *I–V* curve, as shown in Figure 2a, and it closely resembles ferroelectric systems. This phenomenon resembles the characteristics of ferroelectric dipolar relaxation and has also been reported in several recent studies on oxide‐based nonferroelectric materials. In such cases, the behavior is known to be caused by field‐induced dipoles.^[^ ^29^, ^35^, ^39^, ^40^ ^]^ As shown in Figure S4 (Supporting Information), the current measured at 0 V after applying a +7 V or –7 V DC sweep exhibits a clear polarity‐dependent response. A negative current appears after a +7 V sweep, whereas a positive current is observed after a –7 V sweep; in both cases, the current gradually decays toward zero over time. This indicates the presence of a transient and reversible internal electric field that briefly remains after the removal of an external bias. Such polarity‐dependent behavior is consistent with the alignment of field‐induced dipoles, and their orientation is determined by the polarity of the preceding stimulus. This dipolar state is short‐lived and gradually relaxes back to equilibrium, without forming long‐term domains.

To gain deeper insight into the switching behavior of Hf‐72, we also conducted piezoresponse force microscopy analysis on three representative samples (ITO glass, Hf‐18, Hf‐45, and Hf‐72) under identical measurement conditions, as shown in Figure S5 (Supporting Information). Only the Hf‐72 device shows a clear phase transition with stable S‐shaped phase–voltage loops in response to the applied DC bias. In addition, the shape of the amplitude–voltage loop gradually changed as the measuring periods increased, which can be interpreted as a characteristic of FDS.^[^ ^41^ ^]^ A more detailed discussion is provided in Note S5 (Supporting information).

Consequently, the 72 nm condition was found to be the best among the tested thicknesses because it produced the most stable and distinct bimodal switching characteristics, which are essential for dependable RC operation. For future integration and wider applicability, it is essential to guarantee device operational stability and reproducibility. Ensuring device reproducibility and operational stability is critical for broader applicability and future integration. To this end, both cycle‐to‐cycle (C2C) and device‐to‐device (D2D) stability assessments were conducted. The Hf‐72 device maintained consistent ABRS behavior over 100 consecutive cycles under DC voltage sweeps, as shown in Figure 2b. In addition, all 25 Hf‐72 devices consistently display ABRS behavior, according to the D2D results, which are shown in Figure S6 (Supporting Information). As shown in Figure S7 (Supporting Information), each device's C2C stability, together with the statistical analysis of 25 devices, including the distributions of on‐state and off‐state currents with error bars, confirmed excellent reproducibility and reliable switching performance over multiple cycles.

A variety of physical characterization methods were used in order to further clarify the cause of the observed switching behavior. The crystallinity and oxygen defect distribution within the HfO_2_ active layers of each device were examined using X‐ray diffraction (XRD), atomic force microscopy (AFM), and X‐ray photoelectron spectroscopy (XPS) analyses. Figure S8 (Supporting Information) provides a summary of the findings. A shift from an amorphous to a partially crystalline structure is indicated by these analyses, which show a progressive improvement in the atomic ordering from Hf‐18 to Hf‐45 to Hf‐72. At the same time, the oxygen defect density gradually decreased. In particular, Hf‐18 had a relatively smooth and dense surface, an amorphous morphology with few diffraction features, and a high concentration of oxygen vacancies. These structural and chemical transitions were identified as the key factors that drive the evolution from conventional filamentary, which is a nonvolatile switching to volatile bimodal behavior.^[^ ^42^, ^43^, ^44^, ^45^ ^]^ Further discussion is provided in Note S8 (Supporting Information).

The FDS mechanism in Hf‐72 devices is schematically depicted in Figure 2c. Because of the large grain size, low concentration of oxygen defects, and significant film thickness in this system, oxygen ions are suppressed from freely migrating under an applied electric field. Rather, they can only shift from the equilibrium state to the metastable sites in localized areas, which causes electric dipoles to form. The dipoles start to form and produce an internal electric field when the external electric field is applied (Step 1). The internal field progressively approaches a critical threshold where it successfully cancels out the external field as the applied voltage rises (Step 2). The switching cycle is completed when the voltage polarity is reversed, which involves a similar procedure in the opposite direction (Steps 3 and 4).

On the other hand, because of its low crystallinity and high density of oxygen vacancies, the Hf‐18 device shows typical filamentary switching behavior. Oxygen ions are easily displaced from their equilibrium positions due to the high defect density and poor crystallinity, as shown in Figure S9 (Supporting Information) and discussed in Note S9 (Supporting Information). Hf‐45 devices exhibit a hybrid response: they follow the FDS mechanism at low voltages but may display filamentary switching at higher voltages.

This defect‐suppressed environment also affects the current transport characteristics. As shown in Figure S10 (Supporting Information), the Hf‐18 device, when replotted on a double logarithmic scale, exhibits ohmic conduction in the low‐voltage region of the positive voltage sweep (set process), followed by an increasing slope with voltage, indicative of a typical trap‐controlled space‐charge‐limited current (SCLC) mechanism.^[^ ^46^ ^]^ In the negative voltage region (reset process), ohmic behavior is observed throughout, suggesting a defect‐mediated filamentary conduction pathway. In contrast, the Hf‐72 device shows a conduction behavior in which ln(I) is proportional to V in both positive and negative voltage regions, indicating that electron tunneling dominates under reduced‐defect conditions where the migration of oxygen ions is significantly suppressed.^[^ ^35^, ^47^ ^]^ Meanwhile, the Hf‐45 device exhibited tunneling‐dominated conduction at low voltages similar to Hf‐72, but at higher voltages, the current became unstable and showed a proportionality of ln(I) to V^1/2^, indicating a transition to Schottky emission. This instability arises from its thinner film, lower crystallinity, and higher defect density, which lowers the effective barrier height compared to the more stable tunneling in Hf‐72. These observations are consistent with the discussions presented earlier.

**Figure 3:**
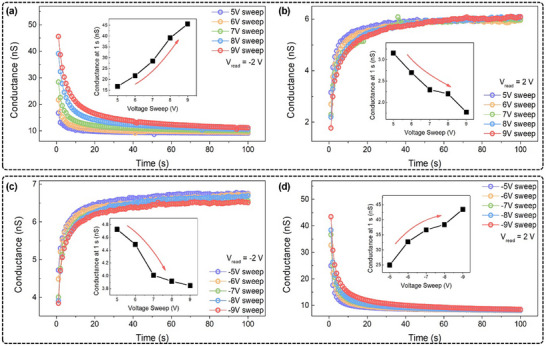
Retention characteristics of the Hf‐72 device monitored for 100 s following a DC sweep. a) Positive‐polarity DC sweep followed by negative read voltage. b) Positive‐polarity DC sweep followed by positive read voltage. c) Negative‐polarity DC sweep followed by negative read voltage. d) Negative‐polarity DC sweep followed by positive read voltage.

Considering the results discussed above, among the three, Hf‐72 exhibits the most stable FDS behavior and its intrinsic field‐induced dipole relaxation dynamics provide key advantages for neuromorphic applications, particularly in RC systems. After a single DC voltage sweep, we observed the conductance state of the Hf‐72 device for ≈100 s in order to assess the field‐induced dipole‐based relaxation dynamics. Investigating the effects of input signal magnitude on the degree of dipole response and subsequent relaxation behavior was another goal of this evaluation. In particular, a unidirectional DC sweep from 5 to 9 V was applied to the device, as shown in **Figure** **3**a,b. Both positive (V_read_ = +2 V) and negative (V_read_ = –2 V) read voltages were then used in order to track the device state's temporal evolution. As seen in Figure 3c,d, the same characterization was carried out again under negative DC sweep conditions (‐5 to ‐9 V). The device's intrinsic field‐induced dipole relaxation behavior was confirmed in each instance by its gradual return to equilibrium. Interestingly, a stronger dipolar state was reflected in the relaxation time as the input voltage amplitudes increased. Stronger stimuli produce longer‐lasting responses, whereas weaker inputs fade more quickly.^[^ ^48^, ^49^ ^]^ These results highlight the Hf‐72 device's suitability for neuromorphic systems by indicating that it can encode input signal strength through its relaxation dynamics. Remarkably, after a negative sweep, an inverse relaxation trend was noted under negative readout conditions. Initially, the internal dipole‐induced field reduced conductivity, but as this field decayed over time, the conductivity gradually increased. A similar phenomenon was observed under a positive sweep followed by a bipolar readout, further confirming the device's reliable bimodal switching, an essential feature distinguishing it from conventional unidirectional neuromorphic RRAM devices. A detailed discussion on the dipolar behavior under various readout polarities can be found in Note S11 and Figure S11 (Supporting Information). Insets in each panel of Figure 3 display the initial current measured immediately after each DC sweep as a function of the sweep voltage amplitude. When the sweep and read voltages share the same polarity, the initial conductivity decreases with increasing voltage. In contrast, when the polarities are opposite, conductivity increases with sweep amplitude. These trends are consistent with the device's operation via the FDS mechanism.^[^ ^50^ ^]^

In addition, we examined how the relaxation behavior evolves with temperature, as shown in Figure S12 (Supporting Information). Under identical sweep amplitudes, higher temperatures led to noticeably prolonged relaxation dynamics. This pattern is probably caused by the increased mobility of oxygen ions at higher temperatures, which encourages the development of dipoles that are stronger or have a greater range. The device takes longer to reach its equilibrium state as a result. These results highlight their importance in dynamic state retention and provide additional information about the thermally activated features of field‐induced dipole relaxation. A detailed discussion is provided in Note S12 (Supporting Information).

In order to implement synaptic functionalities in neuromorphic systems, a device must be able to undergo incremental state transitions in response to the cumulative effect of input stimuli.^[^ ^51^ ^]^ In addition, low‐power operation has become a critical design requirement, making the use of pulse‐driven excitation schemes indispensable.^[^ ^52^ ^]^ The dynamic modulation of conductance in response to tailored pulse inputs enables the optimized emulation of neuromorphic behavior.^[^ ^53^ ^]^ In order to assess the Hf‐72 device's short‐term synaptic properties, we first tested its capacity to simulate synaptic depression and potentiation under repeated pulse inputs without requiring an explicit reset operation. The Hf‐72 device naturally returns to its baseline conductance via spontaneous relaxation, which enables the use of read pulses as implicit reset triggers. This is in contrast to traditional memristors, which frequently need specific reset pulses to restore the initial state. During cycles, this mechanism drastically lowers energy consumption.^[^ ^26^ ^]^ We applied 30 consecutive potentiation and depression pulses per cycle in order to illustrate conductance modulation. To track conductance changes, the read pulses (±2 V) were given after the depression pulses (±2 V, 0.4 ms) and the potentiation pulses (from ±5 to ±9 V, 0.8 ms) were delivered alternately. The device demonstrates symmetrical and smooth potentiation–depression behavior over several cycles under both positive and negative polarities, as illustrated in Figure S13 (Supporting Information). Interestingly, this response is consistent with the relaxation trends previously mentioned, which show that the polarity of the applied pulses determines the direction of the conductance change. These findings further support the Hf‐72 device's suitability for low‐power neuromorphic applications by confirming its ability to implement polarity‐controlled and gradual synaptic weight updates.

We also investigated paired‐pulse facilitation (PPF). We applied voltage pulses with amplitudes between +5 and +9 V and −5 and −9 V. **Figure** **4**a,b shows representative temporal responses to pulse pairs of +5 and −5 V, and Figure 4c,d summarizes the corresponding PPF indices. In our device, two consecutive pulses of the same polarity strengthen the synapse by increasing the field‐induced dipole alignment. However, the resulting built‐in field partially opposes a same‐polarity read field, so the measured read current can decrease even though potentiation is occurring. To avoid misinterpreting this polarity‐dependent readout as synaptic depression, the PPF index is defined by the following Equation (2):

(2) $$\mathrm{PPF}\left(\%\right)=\frac{\left|I_{2\mathrm{nd}}-I_{1\mathrm{st}}\right|}{I_{1\mathrm{st}}}\times 100\%$$

Figure 4Synaptic dynamics and response diversity of the Hf‐72 device under various pulse and readout configurations. a,b) Paired‐pulse input sequences consisting of two consecutive (a) positive or (b) negative voltage pulses. c,d) PPF index as a function of pulse amplitude and inter‐pulse interval under (c) positive‐polarity and (d) negative‐polarity stimulation, demonstrating short‐term synaptic plasticity. Time‐resolved current responses and corresponding Δ*I* (= I_2_ – I_1_) values under input and read pulses of opposite polarity: e,g) current traces and f,h) Δ*I* values across different pulse amplitudes. Time‐resolved current responses and ΔI values under input and read pulses of the same polarity: i,k) current traces and j,l) Δ*I* values. Cumulative evolution of signed conductance (*G*_signed_) following 1–10 consecutive unipolar pulses: m,n) after negative pulses followed by a read voltage of +2 or –2 V, respectively; o,p) after positive pulses followed by a read voltage of +2 or –2 V, respectively.

**Figure:**
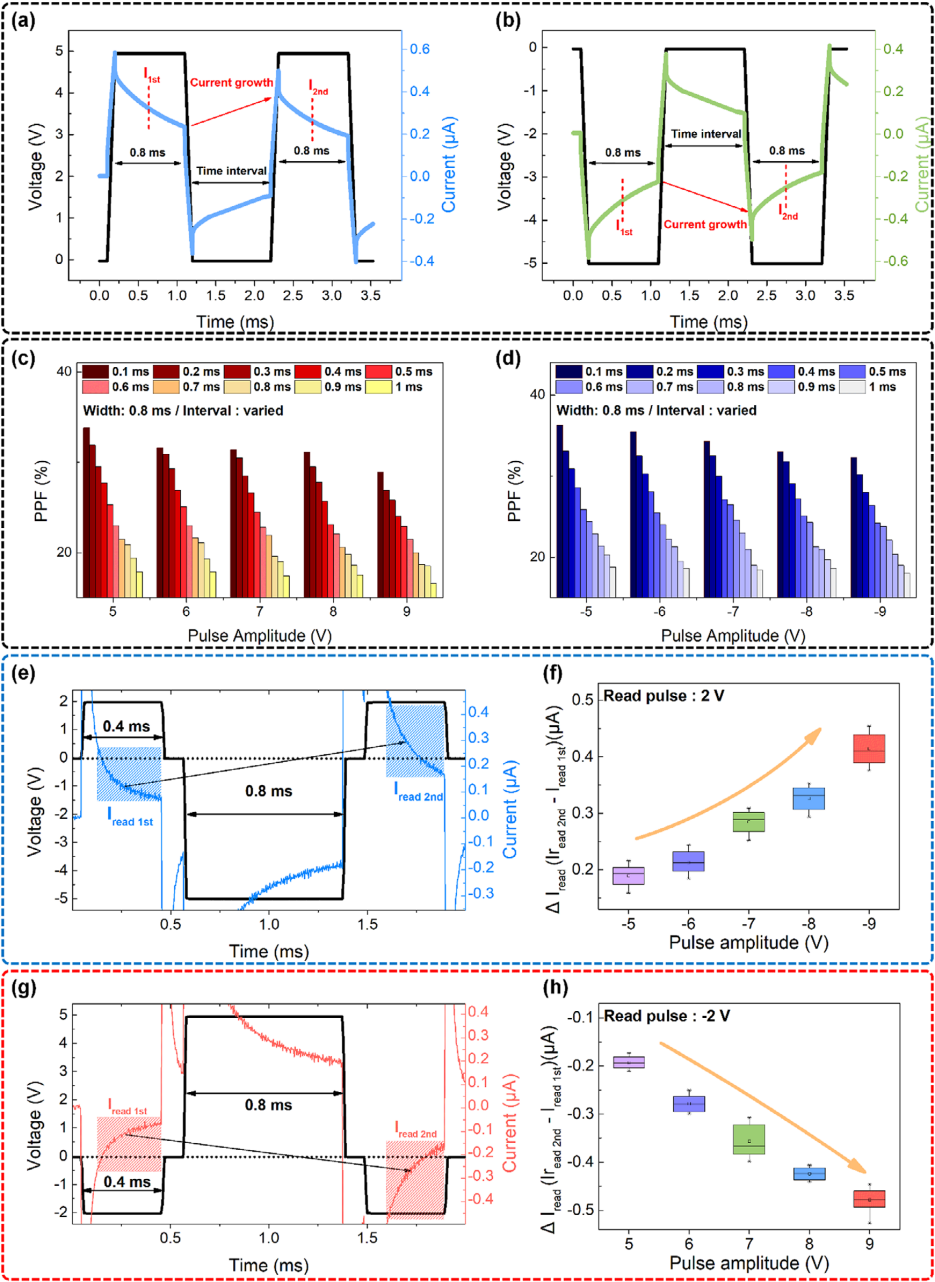

**Figure:**
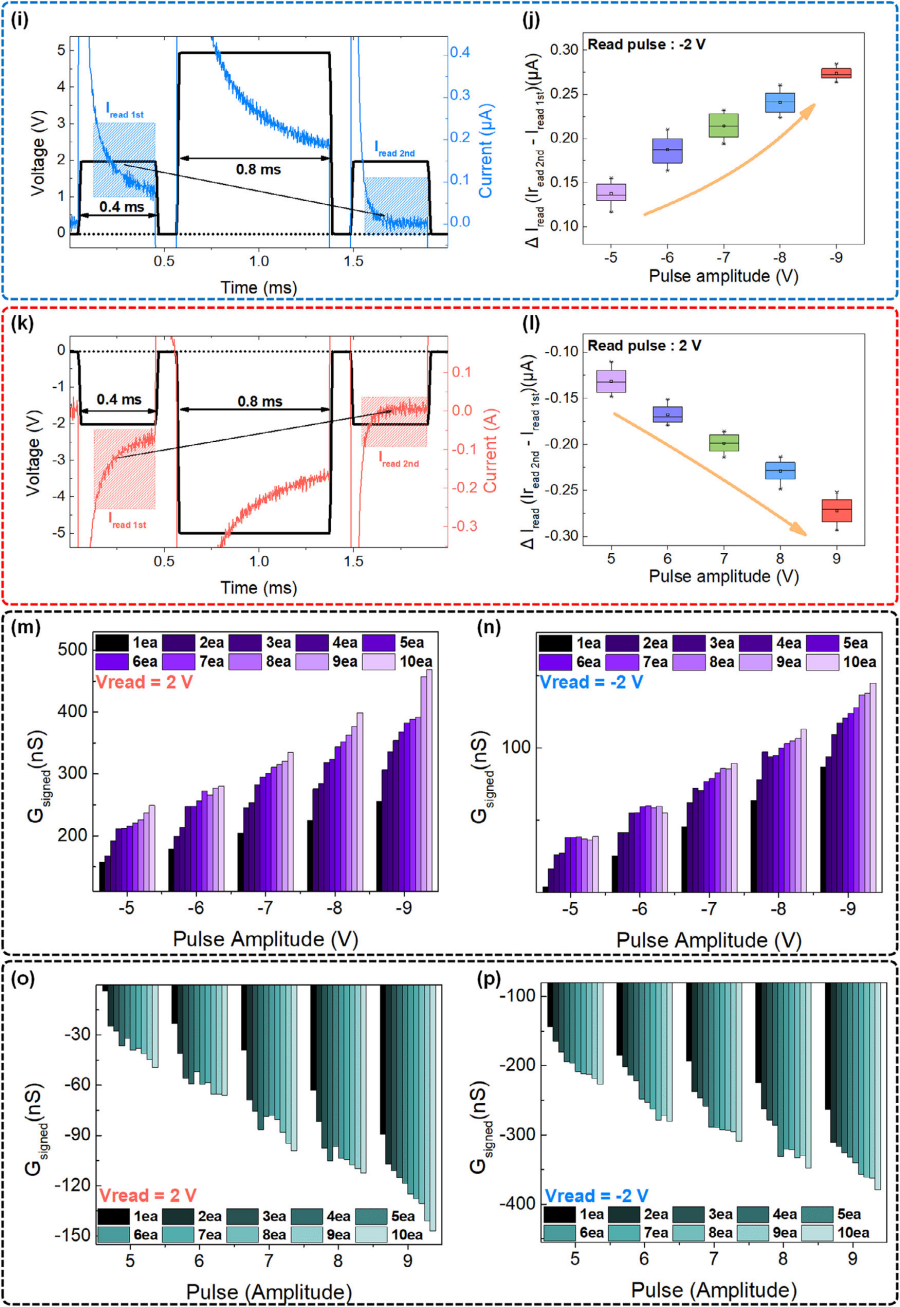

The parameters *I_1st_* and *I_2nd_* represent the currents measured at the center of the first and second voltage pulses, respectively. The pulse intervals were varied from 0.1 to 1.0 ms in 0.1 ms increments. As shown in Figure 4c,d, the PPF index decreases monotonically with an increasing pulse interval, regardless of the pulse polarity. This behavior is attributed to the relaxation of the field‐induced dipole that is generated by the first pulse, which transiently suppresses conductance via an internal electric field. As the interval increases, the dipole partially relaxes, allowing partial recovery of conductance at the time of the second pulse. Consequently, the conductance state during the second pulse is sensitively modulated by the degree of dipole relaxation, which renders the PPF index highly dependent on the pulse interval. Notably, the PPF index was higher at lower pulse amplitudes for a fixed interval, due to the limited nucleation and partial saturation of the internal electric field at higher pulse voltages, which weakens the dipole‐driven modulation during the second pulse.^[^ ^54^ ^]^

To further investigate polarity‐dependent synaptic dynamics, the change in read current (Δ*I* = *I_2nd_* – *I_1st_*) was quantified under different combinations of input pulse polarity (0.8 ms width) and read voltage polarity (±2 V, 0.4 ms width), as shown in Figure 4e–l. Each condition was repeated ten times in order to ensure statistical reliability. As seen in Figure 4e,g, when the input pulse and read voltage had opposite polarities, the second read current increased, reflecting a constructive superposition of the internal and external fields. This effect was more pronounced at higher pulse amplitudes, as shown in Figure 4f,h. Conversely, when the input pulse and read voltage shared the same polarity, the second read current decreased or even reversed under higher pulse amplitudes, as shown in Figure 4i–l. The rapid current suppression or the inversion results from the destructive interference between the read field and the strong internal field created by the dipole. These results show the fast, voltage‐sensitive, and polarity‐dependent switching dynamics of field‐induced dipole‐based mechanisms in Hf‐72. To further quantify the directionally modulated conductance, we defined *G*_signed_ as a signed metric of conductance measured under a bipolar read voltage, which preserved the directionality of the current induced by the field‐induced dipole. Unlike conventional conductance, *G*_signed_ captures both the magnitude and sign of current flow. Figure 4m,n show the evolution of *G*_signed_ under repeated negative input pulses with positive and negative read voltages, respectively, whereas Figure 4o,p show the corresponding behavior for positive pulses. In all cases, *G*_signed_ accumulates incrementally with the number of pulses, with the direction of accumulation determined by the polarity relationship between input and read voltages. *G*_signed_ accumulation occurs in the direction of the read voltage when the pulse and read voltages have opposite polarities because the internal field‐induced dipole strengthens the read field. On the other hand, same‐polarity conditions produce *G*_signed_ accumulation in the opposite direction of the read polarity, which resulted in antagonistic effects. All of these findings support the idea that the Hf‐72 device functions as an analog and polarity‐sensitive artificial synapse that can modulate conductance gradually and plastically. Unlike traditional unidirectional memristors used in RC, its bipolar, bimodal response allows for different outputs under identical input conditions, depending on the read polarity. This input‐output asymmetry highlights the device's potential for next‐generation neuromorphic computing architectures by accurately simulating the nonlinear and multiresponsive dynamics of biological synapses.

**Figure 5:**
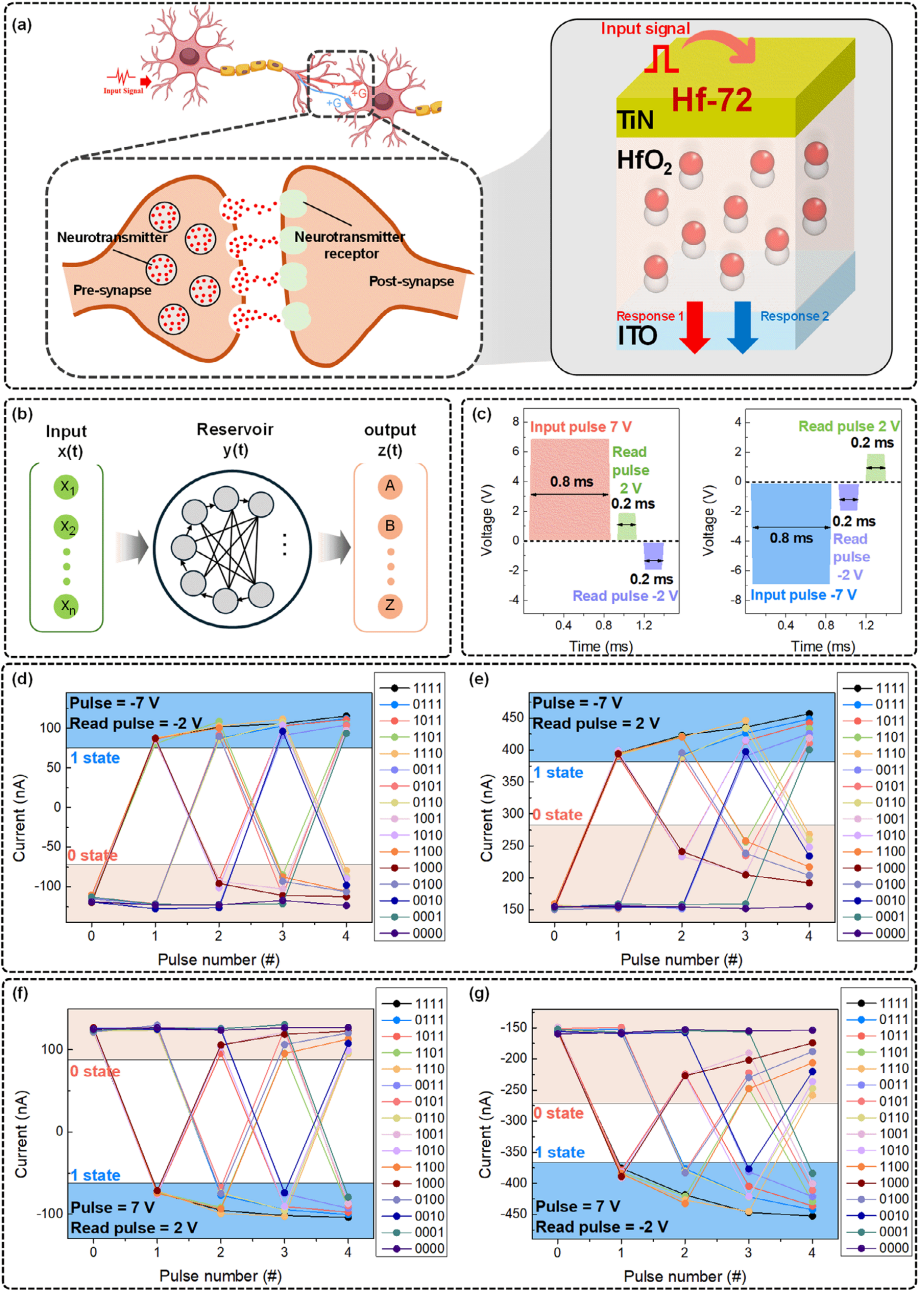
Demonstration of polarity‐sensitive multistate conductance modulation in an Hf‐72‐based reservoir computing system. a) Conceptual illustration of a biological synapse compared with an Hf‐72‐based artificial synapse that produces multiple conductance states from a single input. The biological synapse illustration is partially adapted from a design by brgfx / Freepik. b) Reservoir computing architecture consisting of an input layer, a nonlinear dynamic reservoir layer (Hf‐72 device array), and a readout layer. c) Pulse encoding scheme used to represent the binary state "1," applied as either +7 or –7 V input pulses. Each input is followed by two read pulses (+2 and –2 V, 0.2 ms) to capture bidirectional conductance responses. Conductance response curves for all 16 4‐bit binary input patterns ([0000] to [1111]) under different combinations of input and read polarity: d) –7 V input with –2 V _read_, e) –7 V input with +2 V_read_, f) +7 V input with +2 V_read_, g) +7 V input with –2 V_read_.

The ability of neurons to alter synaptic weights by building up memory traces is a major factor in the efficacy of biological synaptic plasticity. As schematically depicted in **Figure** **5**a, a biological neuron is composed of pre‐synaptic and post‐synaptic neurons, a nucleus, dendrites, and axons. Together, these parts integrate and transmit signals. Compared to traditional unidirectional response devices, a synaptic structure that can generate two output states from a single input significantly boosts the information processing capacity. This bidirectional behavior enables the implementation of complex signal processing and dynamic synaptic weight modulation. The proposed Hf‐72 device operates as an STM element, capable of encoding multiple current states within a single cell, due to its conductance being modulated by both input signal characteristics and readout polarity. Figure 5b presents a typical RC architecture, consisting of two core components: a reservoir layer, comprising recurrently connected nodes that nonlinearly project inputs into a high‐dimensional state space; and a readout layer that linearly maps the dynamic reservoir states to the output signal *z(t)*. To verify the feasibility of integrating the Hf‐72 device within such systems, we experimentally monitored its conductance changes in response to time‐varying inputs. To quantitatively assess the device's state‐tunable characteristics, we applied a sequence of 16 unique 4‐bit binary input codes, ranging from [0000] to [1111], to the Hf‐72 device. The pulse condition used to encode the binary ′1′ is shown in Figure 5c. Each input pulse had an amplitude of either +7 or –7 V and a duration of 0.8 ms, which depended on the experimental condition. Following each input pulse, two read voltages of +2 and –2 V (0.2 ms width) were sequentially applied in order to probe the conductance states under both readout polarities. Pulse duration was optimized in order to guarantee the precise acquisition of the multichannel conductance states and to stabilize the dipole relaxation process. It is crucial to note that each input pulse is followed by two polarity‐specific read operations, which enables the extraction of multiple output features from a single write cycle. This method effectively doubles the informational throughput per pulse and enhances the reservoir system's overall efficiency. Surprisingly, under negative input conditions, the device consistently displayed multistate behavior, producing distinct conductance states for each of the 16 input patterns, as shown in Figure 5d,e. Additionally, as shown in Figure 5f,g, comparable performance was attained under positive input pulses, demonstrating the device's capacity to support multichannel encoding from a single input cell. In summary, the Hf‐72 device demonstrates comprehensive emulation of essential synaptic functions. To further underscore the extent of our characterization, Table S1 (Supporting Information) presents a quantitative comparison with recent memristor‐based neuromorphic systems, confirming the multifunctional superiority of our approach. Furthermore, we estimated the energy consumption per synaptic event for the Hf‐72 device under typical operating conditions (±7 V, 0.8 ms pulse width, and ≈0.31–0.32 µA current), which yielded ≈1.74 nJ (positive) and 1.79 nJ (negative). These findings demonstrate the Hf‐72 device's potential for effective and scalable neuromorphic system integration by confirming that it provides competitive or a better performance in terms of low‐current operation, energy efficiency, and temporal dynamics, as detailed in Note S14, Figure S14, and Table S2 (Supporting Information). The Hf‐72 device's bidirectional and polarity‐sensitive memory properties are confirmed by these results, which highlight its applicability for sophisticated neuromorphic computing, such as especially in RC platforms.

**Figure 6:**
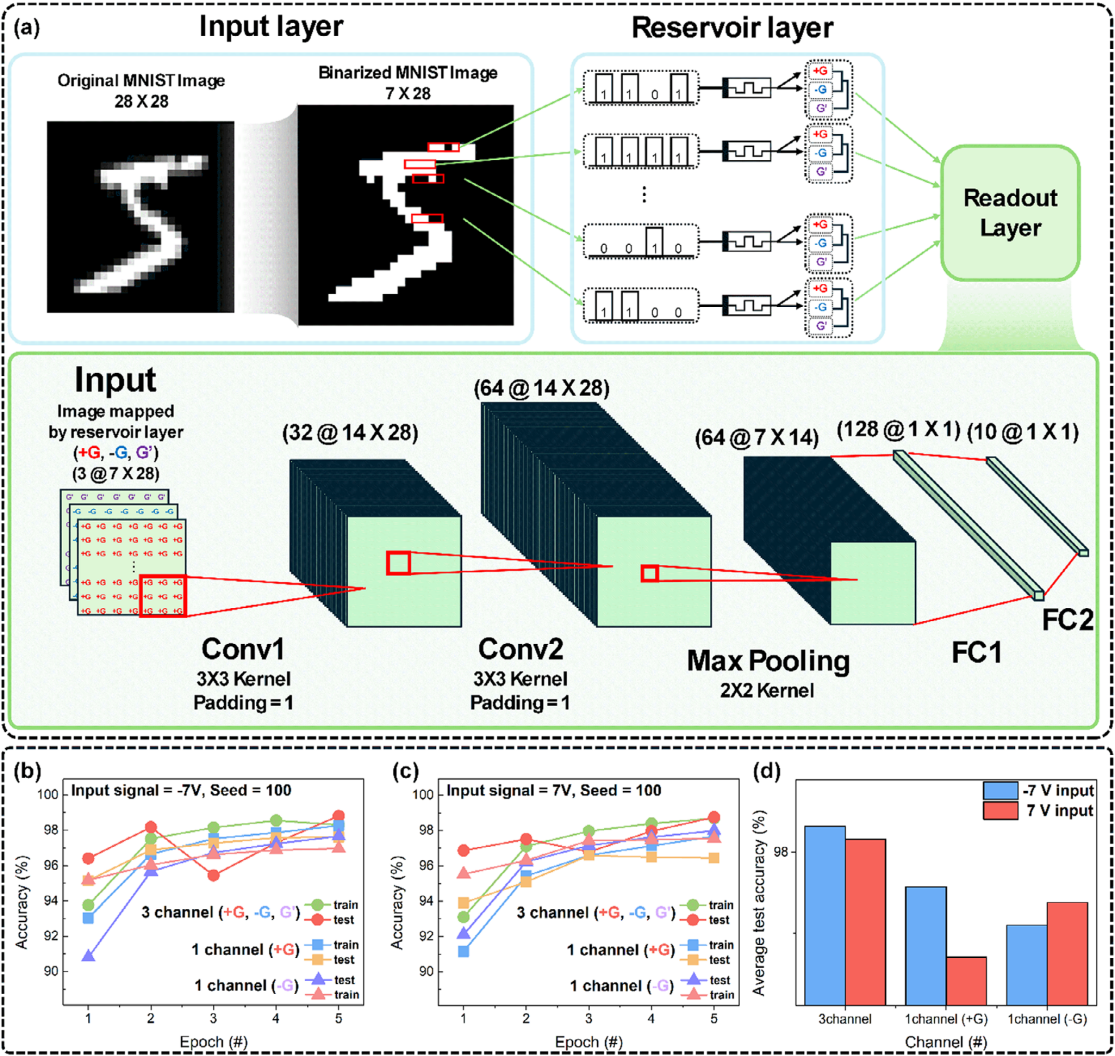
Classification performance of bimodal memristor‐based RC framework. a) Schematic of the RC simulation framework for image classification based on Hf‐72. b,c) Training and testing accuracy over 5 epochs with seed = 100 for (b) negative (–7 V) and (c) positive (+7 V) input signals, respectively. d) Averaged test accuracy across 6 random seeds (0, 1, 2, 3, 42, and 100).

Lastly, we conducted an image classification modeling experiment using a reservoir computing (RC) system based on the Hf‐72 memristor in order to assess the computational applicability of the suggested device. As shown in **Figure** **6**a, the simulation framework consists of three sequential layers: input, reservoir, and output. After receiving raw data, the input layer acts as the model's entry point and sends the data to the reservoir layer. The core of the RC architecture is the reservoir layer, which projects inputs into a high‐dimensional space for feature extraction through nonlinear transformations. Its design is crucial for system performance. The output layer subsequently maps these extracted features to task‐specific predictions. For effective operation, the memristive reservoir must exhibit (1) nonlinear (*I–V*) characteristics and (2) an STM characteristic, which allows responses to reflect recent inputs. Both of these requirements are satisfied by the Hf‐72 device. To construct a functional RC system for pattern recognition, we defined three channel responses from the Hf‐72 device: +*G* (read at +2 V), –*G* (read at –2 V), and *G*′, the difference between +G and –G. We were able to project the input into a three‐channel high‐dimensional representation space, which enables richer information encoding and improved data separability, by using these responses to create multiple embeddings from a single input.^[^ ^32^, ^55^, ^56^ ^]^ We used the MNIST handwritten digit dataset, which consists of 10 000 test images and 50 000 training images from the U. S. National Institute of Standards and Technology (NIST) database. High‐dimensional input mapping was conducted by applying electrical pulses to an array of 196 Hf‐72 devices, which acted as the reservoir layer. This was done by taking advantage of the nonlinear and temporally dependent behavior of these devices.^[^ ^57^ ^]^ To guarantee statistical reliability and reproducibility, the training procedure was carried out five times using six distinct random seeds (0, 1, 2, 3, 42, and 100). Both the training and testing progress were analyzed accordingly. Three configurations were compared: 1) using all +*G*, –*G*, and *G*′ channels (three‐channel mode), 2) using only +*G*, and 3) using only –*G*. This comparison allowed us to quantify the impact of the device's bipolar bimodal switching behavior on information representation. All configurations shared the same network architecture except for the number of input channels to the readout layer. Figure 6b,c presents the representative results for Seed = 100. The three‐channel configuration consistently outperformed the single‐polarity cases, achieving over 96% accuracy early in training and exceeding 98% accuracy on the test dataset by the end. Figure 6d shows that the proposed three‐channel configuration consistently achieved the highest classification accuracy across all 6 random seeds. Figures S15 and S16 (Supporting Information) further present the corresponding confusion matrices for the positive and negative input modes, respectively, which reinforced that the bipolar bimodal switching characteristics physically embedded in the Hf‐72 device significantly enrich the information representation capacity of the RC system. Importantly, this three‐channel architecture does not function as a conventional preprocessing step involving duplication or re‐encoding of inputs. Instead, it exploits intrinsic device physics such as asymmetric ion migration, dipole relaxation, and voltage‐polarity‐dependent detection to produce three physically distinct and nonlinear responses (+*G*, –*G*, and *G*′) from a single input pulse. The neural network can extract more complex spatiotemporal features due to these orthogonal responses, which act as varied embeddings that broaden the representational space. This improves learning effectiveness and class separability. In other words, although the reservoir is composed of structurally identical Hf‐72 devices, we achieved functional heterogeneity by leveraging the multimodal dynamic responses of each device. This aligns with the theoretical studies, which demonstrated that heterogeneity among reservoir nodes improves state separability and information encoding.^[^ ^58^ ^]^ However, their proposed structural heterogeneity often has practical limitations, such as more complex fabrication. As a result, calibration needs are specific to the device, which creates challenges with array integration.^[^ ^59^, ^60^ ^]^ The benefits of both homogeneous and heterogeneous reservoir architectures are combined in our scalable and repeatable method, which achieves multimodal functionality in a single device as well while remaining fully compatible with the CMOS process.

Ultimately, to further enhance the completeness of our works, we performed simulations by intentionally adding heterogeneity to the device responses in order to assess the effect of device‐level variability on the RC performance. Excessive heterogeneity can skew the input‐to‐state mapping and impair system performance, as demonstrated by the progressive decline in the classification accuracy with increasing variation ratios, as shown in Figure S17 (Supporting Information). Nevertheless, the experimental measurements of our fabricated Hf‐72 devices, as shown in Figures S6 and S7 (Supporting Information), revealed that the inherent variation of ≈22% across cycles and devices had a negligible effect and the classification accuracy remained above 97%. These findings underscore the importance of consistent temporal encoding and state space separability in preserving our system performance.

These results, in conjunction, demonstrate the Hf‐72 device's great potential as a next‐generation neuromorphic computing platform that can provide both high precision and low power consumption. Specifically, the study's proposed three‐channel readout capability is expected to serve as a basis for future research directions in physical RC and offers promising applicability to a variety of fields, which include high‐dimensional dynamical systems such as chaotic modeling.

## 3 Conclusion

In this work, we report an HfO_2_‐based volatile memristor that enhances information representation at the single‐cell level through field‐induced dipole‐based bimodal switching. By controlling the cumulative energy during RF sputtering, we modulated the crystallinity and oxygen vacancy profile of the HfO_2_ thin film, enabling a transition from conventional filamentary switching to field‐induced dipole‐driven resistive switching. When exposed to electrical stimuli, the optimized device, known as Hf‐72, demonstrates STM behavior, which includes spontaneous relaxation and gradual conductance modulation. The device consistently generated sixteen distinct conductance states under a 4‐bit pulse scheme, and the readout was reliant on the applied voltage's polarity. The available encoding space for information representation is greatly increased by this bipolar switching property. The Hf‐72 memristor demonstrated a classification accuracy of over 98% on the MNIST dataset when it was used as a physical reservoir in an RC framework. These results demonstrate how Hf‐72 can be used as a flexible platform for multichannel encoding and high‐dimensional signal processing in neuromorphic computing systems.

## 4 Experimental Section

### Fabrication Process of the HfO_2_‐Based Memristor

The TiN/HfO_2_/ITO memristor structures, with varying HfO_2_ thicknesses, were fabricated following the process flow outlined in Figure S18 (Supporting Information). Initially, ITO‐coated quartz substrates were sequentially cleaned by immersion in acetone for 10 min, methanol for 10 min, and deionized (DI) water for 10 min. After cleaning, an HfO_2_ layer was deposited onto the ITO‐coated quartz substrates as a resistive switching layer using RF sputtering (KVS‐2000L) at 100 W in an Ar ambient (20 sccm). A metal shadow mask with a circular design was then used to RF sputter a 40 nm‐thick TiN top electrode (TE) in an Ar ambient (18 sccm) and a N_2_ ambient (2 sccm).

### Analysis of the Proposed HfO_2_‐Based RRAM

A number of measurements were used to look at the physical and chemical properties of HfO_2_ films of different thicknesses in order to see how they change with thickness. XRD was used to look at changes in crystallization levels, and AFM was used to look at changes in the surface roughness and piezoelectric hysteresis loops. We also used XPS to look at how the thickness of the HfO_2_ films affected their chemical makeup and material properties. As seen in Figure S19 (Supporting Information), a Keithley 4200 SCS was used to analyze the electrical properties, which included *I–V* curve characteristics, and a 4225‐PMW ultrafast module was used to measure the pulses.

### Implementation Details of the RC System

The RC‐based image classification experiment was conducted using the MNIST handwritten digit dataset, which comprises 50 000 training images and 10 000 test images. An array of 196 Hf‐72 devices, each receiving electrical pulse inputs, was used in order to implement the reservoir layer. A convolutional neural network (CNN) architecture with two convolutional layers, a max‐pooling layer, and a fully connected layer was used for the offline training. The first convolutional layer had three input channels and thirty‐two output channels, with a stride of one and a kernel size of three by three. The second convolutional layer added 64 output channels while maintaining the same kernel size and stride. Zero padding was used in order to preserve spatial dimensions during convolution operations.^[^ ^26^ ^]^ Batch normalization and ReLU activations were used on all of the convolutional layers,^[^ ^61^ ^]^ to make the training more stable and the model more nonlinear. Max pooling was also used to get rid of background noise and highlight important features.^[^ ^62^ ^]^ A fully connected layer with 128 hidden neurons received the output from the convolutional layers. For improved convergence, the network was trained using the Adam optimizer with a Nesterov Accelerated Gradient (NAG).^[^ ^63^ ^]^ Cross‐entropy served as the loss function, and the learning rate was fixed at 0.001. To make sure the results could be repeated and to test how well the model worked, the training process was done five times for each of the six different random seeds (0, 1, 2, 3, 42, and 100).

## Conflict of Interest

The authors declare no conflict of interest.

## Supporting information



Supporting Information

## Data Availability

The data that support the findings of this study are available from the corresponding author upon reasonable request.
