# Supplementary material for: Polarity‐Controlled Volatile HfO2 Memristors with Bimodal Conductance for Neuromorphic Synapses and Reservoir Computing
Source: Adv Sci (Weinh). 2025 Nov 3;13(3):e15926. doi: 10.1002/advs.202515926 (PMC12806217; doi:10.1002/advs.202515926)
Supplement: Supplementary file 1 — Supporting Information [file ADVS-13-e15926-s001.docx]

**Supporting Information**

**Polarity-Controlled Volatile HfO_2_ Memristors with Bimodal Conductance for Neuromorphic Synapses and Reservoir Computing**

**This Supporting Information file includes:**

Note S1, S3, S5, S8, S9, S11, S12, S14

Figure S1 to S19

Table S1, S2

SI References

**Table S1**

| **No** | **Structure** | **Cycle-to-Cycle** | **Device-to-Device** | **Relaxation**  **(# of Condition )** | **Paired-pulse facilitation**  **(# Condition)** | **Potentiation-Depression**  **(# of Condition )** | **# of Reservoir states** | **Polarity dependence** | **Ref** |
| --- | --- | --- | --- | --- | --- | --- | --- | --- | --- |
| 1 | Pt/TaO_x_/TiN | 100 | 30 | 9 | 1 | 1 | 16 | N/A | ^[1]^ |
| 2 | Pt/Ta_2_O_5_/HfO_2_/TiN | 50 | N/A | N/A | N/A | N/A | 16 | N/A | ^[2]^ |
| 3 | ITO/ZrO_x_/TaN | 100 | 10 | 4 | 1 | 5 | 256 | N/A | ^[3]^ |
| 4 | Au/PZT/SFO/SRO//STO | 20 | 15 | 1 | 1 | 15 | 16 | N/A | ^[4]^ |
| 5 | Pd/Au/WO_x_/W | N/A | N/A | N/A | N/A | N/A | 16 | N/A | ^[5]^ |
| 6 | TiN/WO_x_/Pt | 100 | 4 | 1 | 1 | 1 | 16 | N/A | ^[6]^ |
| 7 | TiN/TiO_x_/HfO_x_/Pt | 100 | 9 | 1 | 1 | 1 | 10 | N/A | ^[7]^ |
| 8 | ITO/NiO_x_/A | N/A | N/A | N/A | N/A | N/A | 16 | N/A | ^[8]^ |
| 9 | LIF/Al/PCBM/C H_3_NH_3_PbBr_3_/PED OT:PSS/ITO | N/A | N/A | 3 | 1 | N/A | 10 | N/A | ^[9]^ |
| 10 | ITO/p-NiO/n-IGZO/Ti/Pt | N/A | N/A | 1 | 1 | 7 | 16 | N/A | ^[10]^ |
| 11 | Pt/Ag/ZrO2/TiN | 20 | 10 | 6 | 1 | N/A | 16 | N/A | ^[11]^ |
| 12 | ITO/ZnO/IGZO/ZnO/ITO | 55 | 9 | 1 | 1 | 32 | 16 | N/A | ^[12]^ |
| 13 | TiN/TiO_x_/WO_x_/Pt | 30 | 10 | 6 | 1 | 1 | N/A | N/A | ^[13]^ |
| 14 | CZO/ITO/glass | 100 | 5 | 3 | 1 | 1 | N/A | N/A | ^[14]^ |
| 15 | TiN/HfO_2_/ITO | 100 | 9 | 20 | 10 | 20 | 16 | O | **This work** |

**Table S1.** Quantitative comparison of experimentally evaluated synaptic characteristics in recent synaptic devices.

**Note S1.**

Reservoir computing with memristors is a new way to do math that can handle input signals that change over time. Volatile memristive devices, in particular, are leveraged as hardware platforms for short-term memory due to their inherent ability to temporarily alter conductivity in response to external stimuli and recover to the initial state once the stimulus is removed. These features make volatile memristors great at capturing temporal correlations in input sequences. They are well-suited for many time-series tasks, such as recognizing speech, classifying signals, and finding anomalies.

Recent volatile memristors used in reservoir layers generally operate through two mechanisms: (i) filament-based switching and (ii) non-filament-based switching. Filament-based volatile memristors form conductive paths via oxygen vacancy filaments within metal oxide layers, which revert to low-conductivity states when the filaments dissolve after stimulus removal. However, these devices exhibit irreversible asymmetric conduction dynamics and typically allow only unidirectional conductance modulation. In contrast, non-filament-based volatile memristors rely on ion migration or interface barrier adjustments to modulate conductivity, providing more uniform device properties. However, these devices likewise change their conductivity solely in one direction. Both types of conventional volatile memristors inherently limit information representation due to their unidirectional conductance modulation, which restricts their applicability for complex reservoir computing tasks that require multidimensional encoding.

Figure S1a illustrates the limitations with conventional volatile resistive switching memory (RRAM) devices. Both filament-based and non-filament-based configurations increase conductance in one direction upon stimulation, and they go back to their initial states when the stimulus is removed. This restriction limits information encoding diversity and expressiveness when used as reservoir layers. Figure S1b, on the other hand, shows the conceptual design of the proposed TiN/HfO_2_/ITO volatile memristor, which can switch in both directions based on field-induced dipoles. This device shows bimodal responses, meaning that conductivity can either increase (+G) or decrease (−G) depending on the read polarity. This lets you read the conductance in both ways at the same time.

The bidirectional modulation is caused by dipolar effects. Under external electric fields, the shift of oxygen ions from the equilibrium positions to the metastable sites induces dipole in localized regions of the HfO_2_ film, with the alignment direction determined by the applied electric field polarity. The polarity of the electric field determines the manner in which the dipoles will line up. When the stimulus is removed, the dipoles gradually dissipate, and conductivity returns to the initial state. The alignment direction can be reversed depending on the applied voltage polarity, and both positive and negative conductivity changes are possible depending on the read polarity. Thus, the device inherently supports bimodal responses, overcoming the unidirectional limitations of conventional volatile memristors without requiring additional post-processing. Consequently, the device can generate a wide range of conductivity states from identical input stimuli, significantly enhancing encoding resolution and multiform information representation within reservoir computing systems.

Figure S1c further elucidates the physical origin of these electrical properties, highlighting the ion bombardment effects observed during radio frequency (RF) sputtering. Repeated collisions between Ar⁺ ions from plasma and the growing HfO_2_ film increase cumulative energy, leading to a partially crystallized HfO_2_ layer characterized by enlarged grains and reduced oxygen vacancies [15]. This microstructure supports field-induced dipole formation under external electric fields and spontaneous dipole relaxation once the stimulus ceases. This dipole-driven volatile switching mechanism is validated by the abnormal butterfly-shaped current–voltage (*I–V*) characteristics.

**Figure S1**


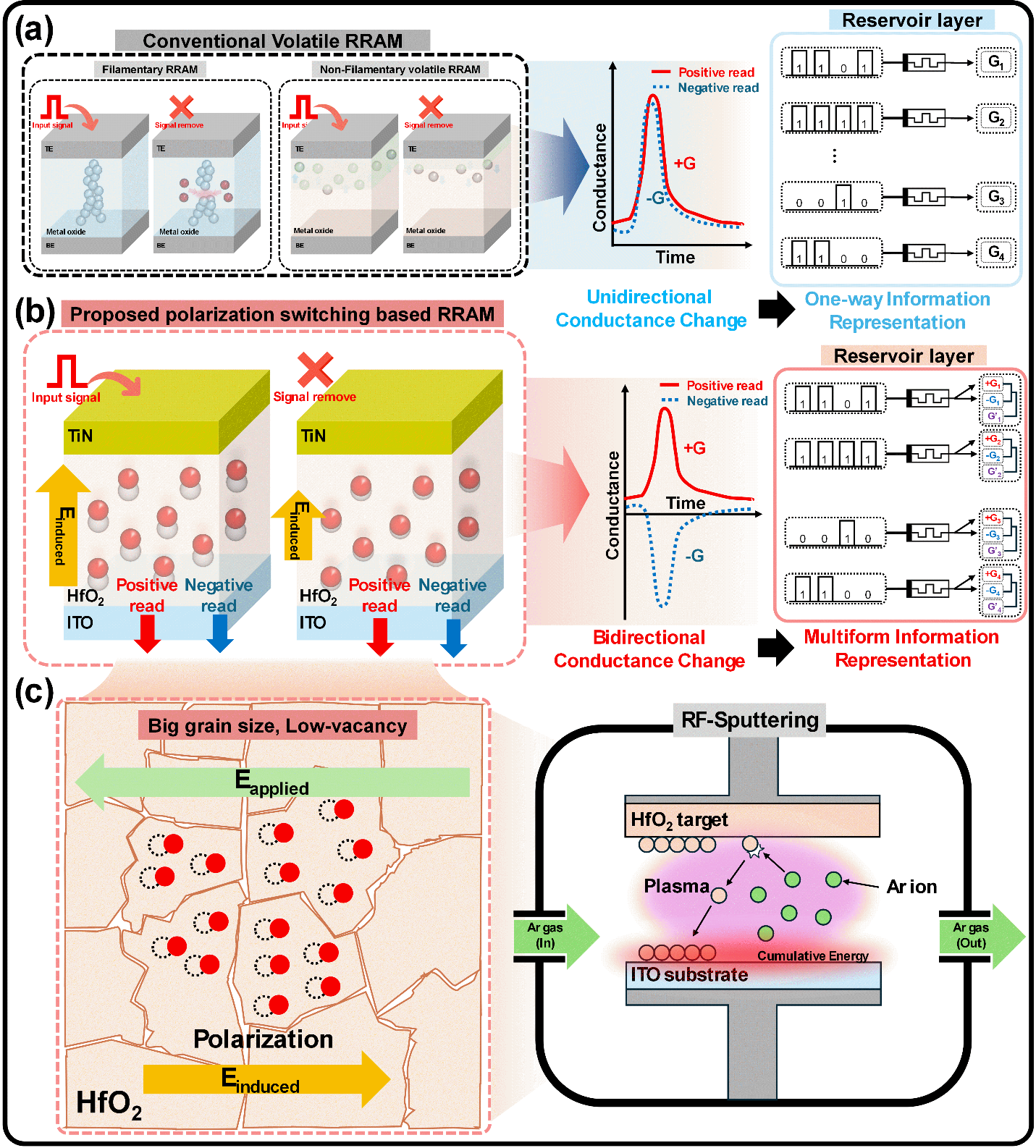


**Figure S1**. Comparison of conventional unidirectional and field-induced dipole-driven bimodal RRAM structures. (a) Schematic of conventional unidirectional volatile RRAM. (b) Schematic of the proposed field-induced dipole switching-based RRAM exhibiting bipolar bimodal conductance changes. (c) Illustration of large grain formation and reduced vacancy concentration in HfO_2_, achieved by controlling cumulative ion energy during RF sputtering.

**Figure S2**


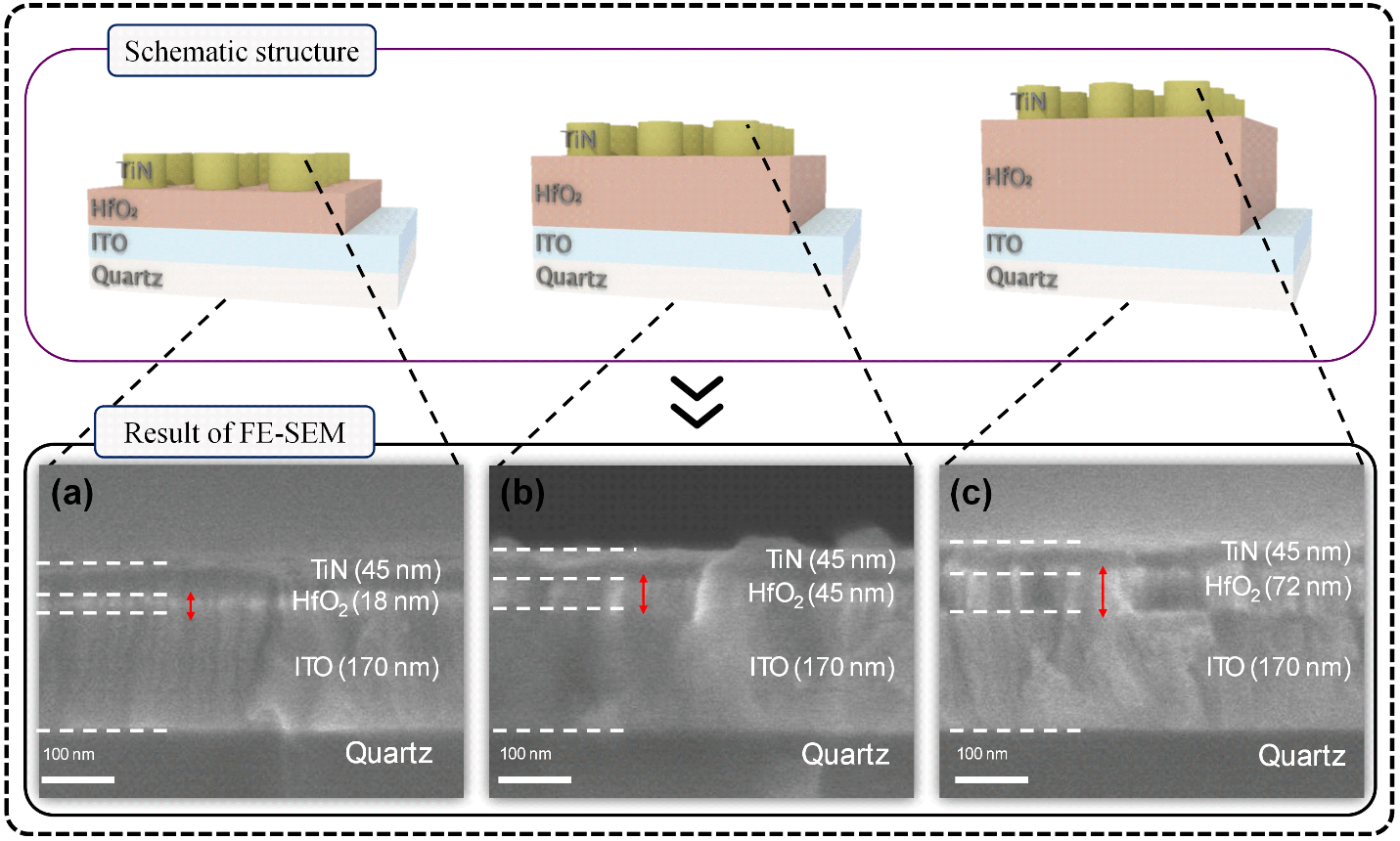


**Figure S2**. Cross-sectional SEM images of each device fabricated under different levels of cumulative sputtering energy. The cumulative energy density increases progressively from (a) to (c).

**Note S3**

Figure S3a shows that the DC voltage sweep applied to the Hf-18 device followed a sequence of 0 → 4 → 0 → –1.5 → 0 V. The forming process occurred around 3.3 V during the initial positive voltage sweep. A RESET phenomenon was subsequently observed at approximately –1 V as the sweep direction was reversed toward negative voltages. This behavior indicates that the device transitioned from the low-resistance state (LRS) to the high-resistance state (HRS) owing to the partial or complete rupture of the conductive filament that was previously formed within the oxide layer.

Furthermore, a positive voltage was reapplied to transition the device back from HRS to LRS, resulting in an SET event at approximately 1 V. This abrupt current increase confirmed the reformation of the conductive filament.

Figure S3b displays the I–V characteristics for the Hf-45 device. The DC voltage sweep followed the sequence 0 → 4 → 0 → –4 → 0 V, which incrementally increased the sweep voltage from an initial ±1 V up to a maximum of ±8 V. The Hf-45 device exhibited gradual resistance changes within a low-voltage range of −4 to −6 V and 4 to 6 V, similar to Hf-72, and demonstrated abnormal bipolar resistive switching (ABRS) characteristics. However, upon entering a higher voltage regime (above ±7 V), oxygen vacancies were no longer confined around equilibrium positions, but they instead gained sufficient mobility in order to move freely within the oxide film. The rapid migration of oxygen vacancies in this regime led to unstable current conditions, eventually causing a sharp rise in the current levels.

Figure S3c shows that the Hf-18 device maintains stable retention characteristic, with a conductance ratio (CR) of approximately 4×10⁴ over 10⁴ seconds under a read voltage of 0.5 V.
Figure S3d demonstrates the endurance behavior of the Hf-18 device over 100 cycles, exhibiting a CR on the order of 10¹. These results indicate that the Hf-18 device operates as a conventional filament-based non-volatile RRAM.

**Figure S3**

**
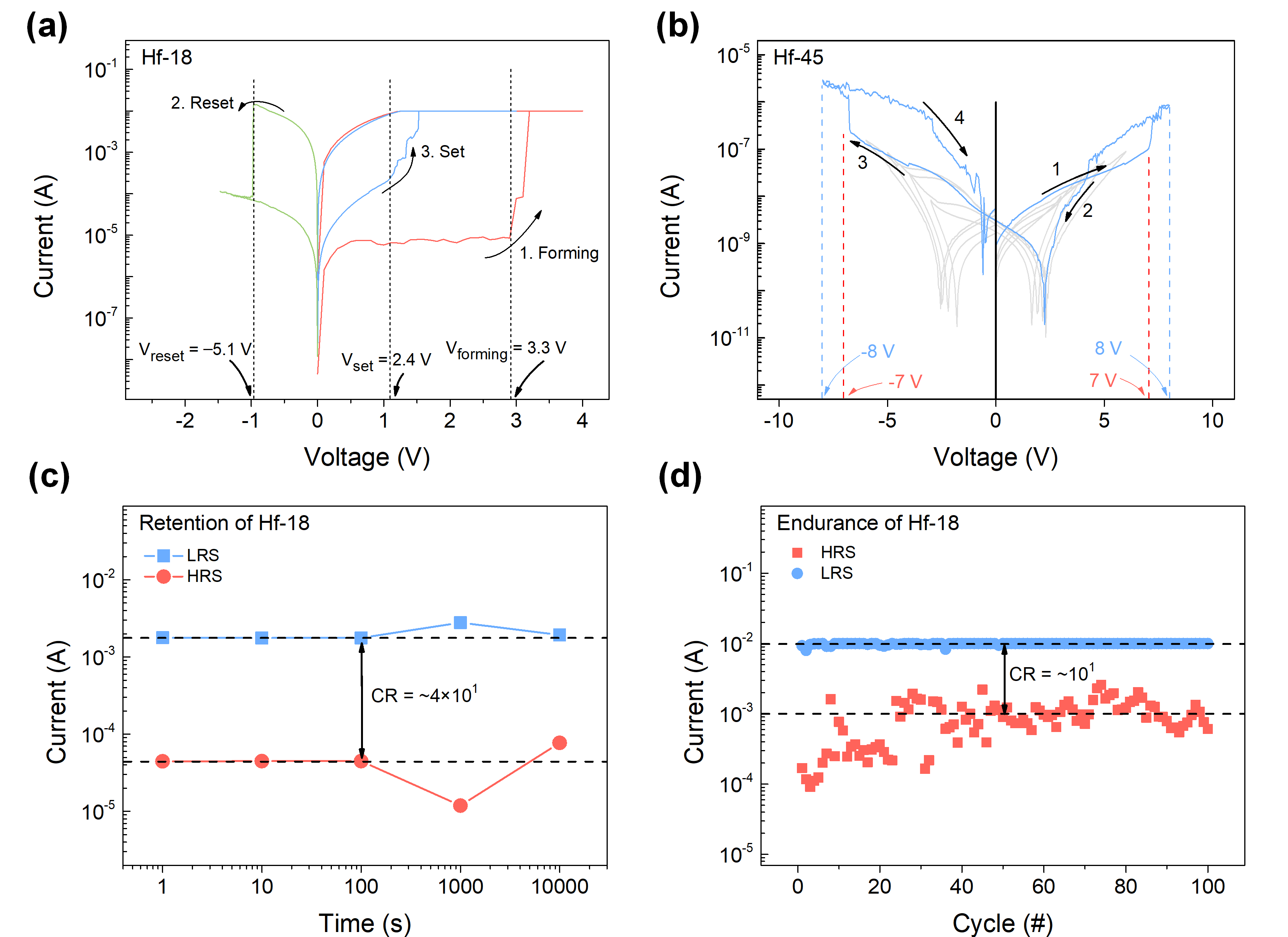
**

**Figure S3**. Electrical characteristics of Hf-18 and Hf-45. *I-V* characteristics of the (a) Hf-18 and (b) Hf-45 device under DC voltage sweeps. (c) Retention characteristics for 10^4^ s and (d) Endurance characteristics for 100 cycles of Hf-18.

**Figure S4**

**
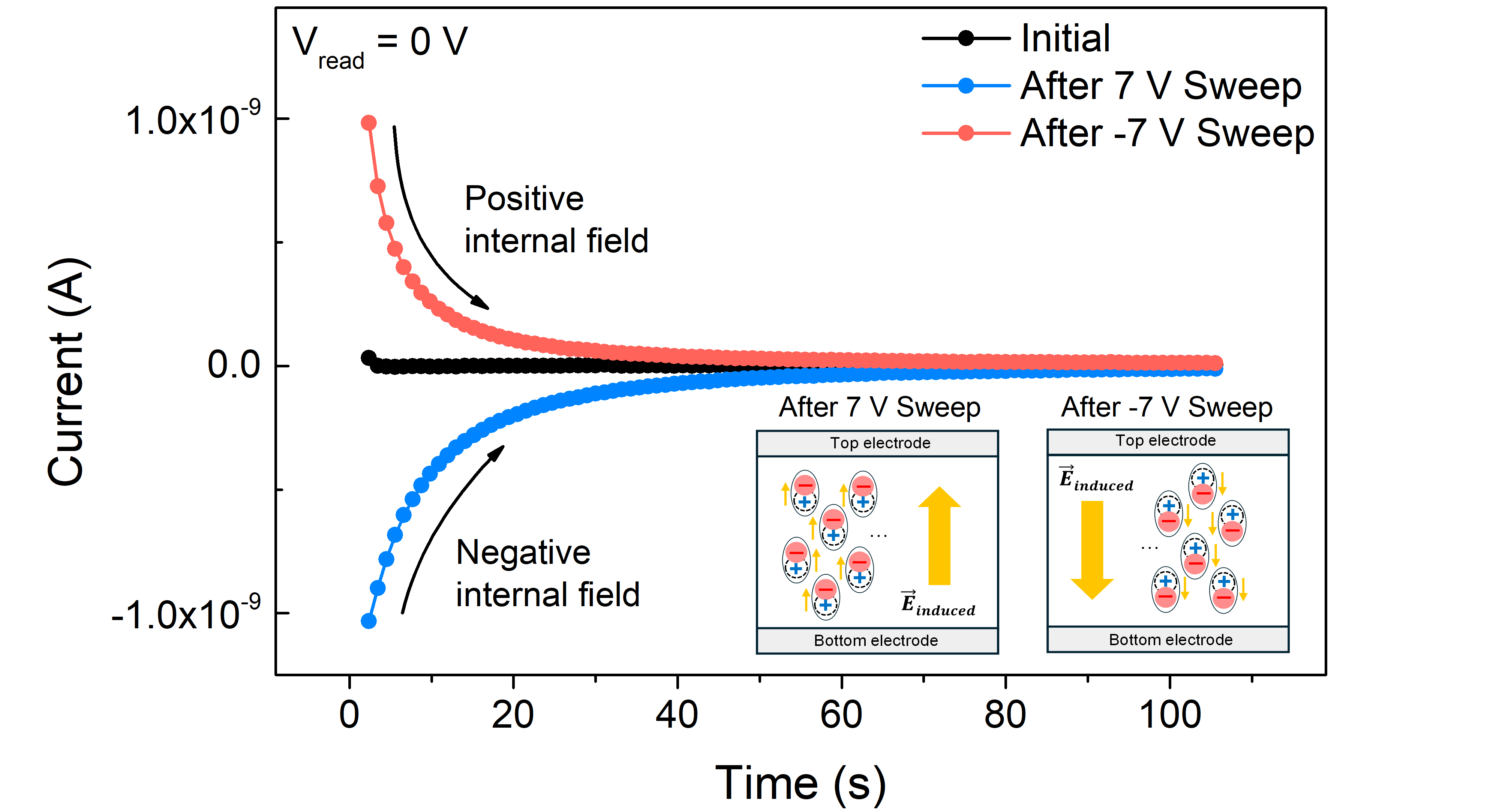
**

**Figure S4.** Zero-bias current decay after ±7 V sweeps, indicating polarity-dependent residual fields from aligned dipoles. Insets illustrate the corresponding dipole orientations.

**Note S5**

We used piezoresponse force microscopy to measure four samples (ITO glass, Hf-18, Hf-45 and Hf-72) under the same DC voltage sweep conditions to determine the origin of the ABRS behavior in Hf-72.

Figure S5a shows that the ITO glass substrate did not show any phase reversal when a DC bias was applied. This confirms the absence of piezoelectric activity in ITO glass, and the effect of electrostatic artifacts was minimized in our measurement setup. Additionally, we couldn't see a clear S-shaped phase-voltage curve was observed for the Hf-18 device as shown in Figure S5b. Instead, based on the applied voltage, we saw irregular and unstable phase changes. This is probably because conductive filament formation causes abrupt changes in the current path.

In contrast, as shown in Figure S5c,d, both the Hf-45 and Hf-72 devices exhibited S-shaped phase–voltage loops, a behavior typically reminiscent of ferroelectricity but, in non-ferroelectric oxides, arising instead from field-induced dipoles ^[16]^.However, when the voltage range was further increased, the loops of the Hf-45 device became increasingly distorted, indicating that the dipoles, which remain in a meta-stable equilibrium at lower fields, deviate from this state under higher energy input. This instability can be attributed to the thinner thickness, higher defect density, and lower crystallinity of Hf-45 compared with Hf-72.

On the other hand, the Hf-72 device maintained clear and stable S-shaped loops even at higher voltages, with the loop width gradually expanding with increasing bias consistent with robust and stable dipole formation. Additionally, the amplitude–voltage loops displayed discernible shape changes with longer measurement periods as shown in Figure S5e, in contrast to conventional ferroelectric materials where the loop shape stays almost constant ^[16]^.

Consequently, the transition from the irregular and unstable phase behavior observed in Hf-18 and Hf-45 to the clear phase transition response in Hf-72, accompanied by its gradually evolving amplitude–voltage curves over longer measurement periods suggests that the ABRS originate from field-induced dipole alignment with rapid relaxation, rather than from remanent ferroelectric polarization.

**Figure S5**

**
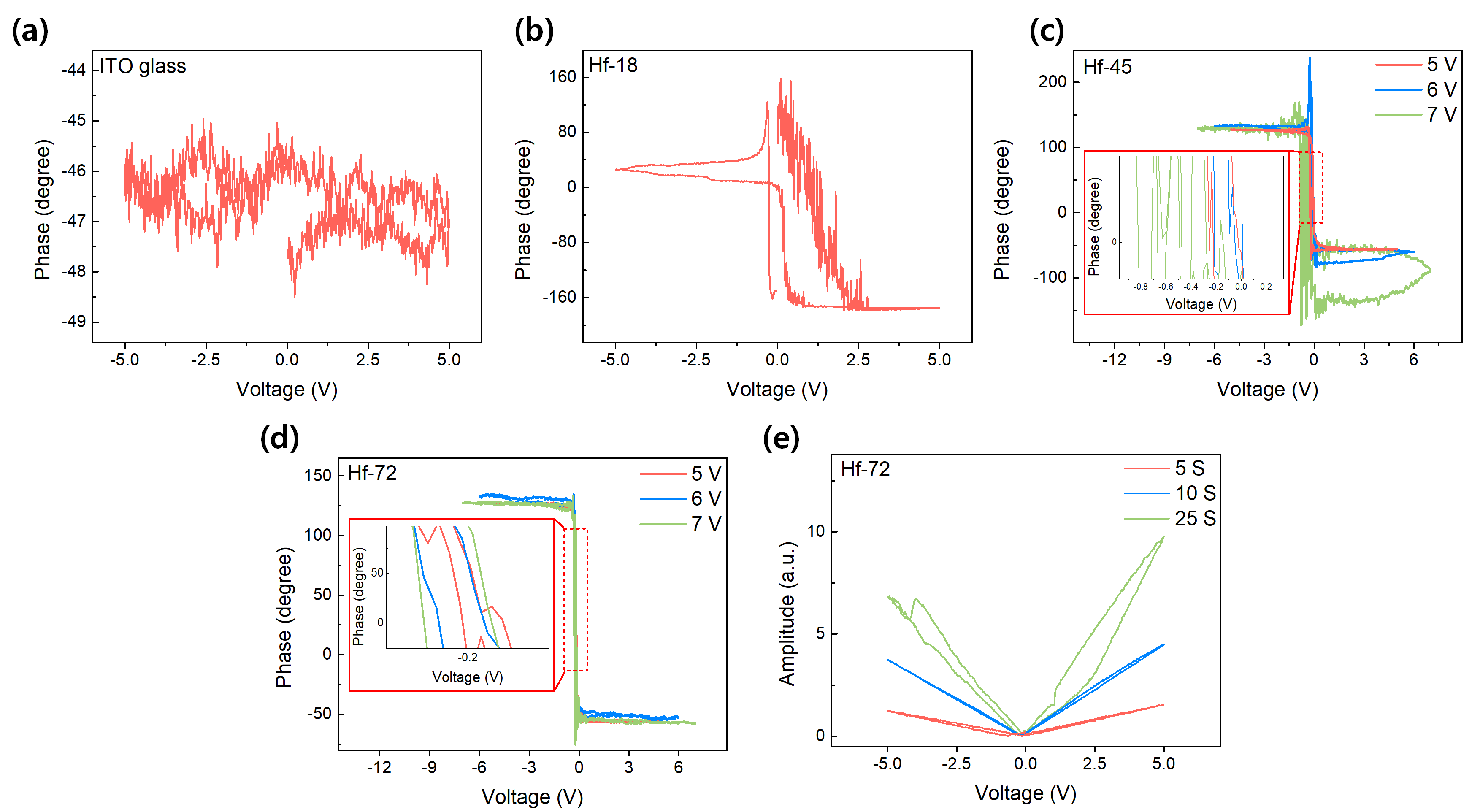
**

**Figure S5**. Phase responses measured by piezoresponse force microscopy under identical DC voltage sweep conditions for (a) ITO glass, (b) Hf-18, (c) Hf-45 and (d) Hf-72 devices. (e) Amplitude–voltage curves of the Hf-72 at different measurement periods

**Figure S6**


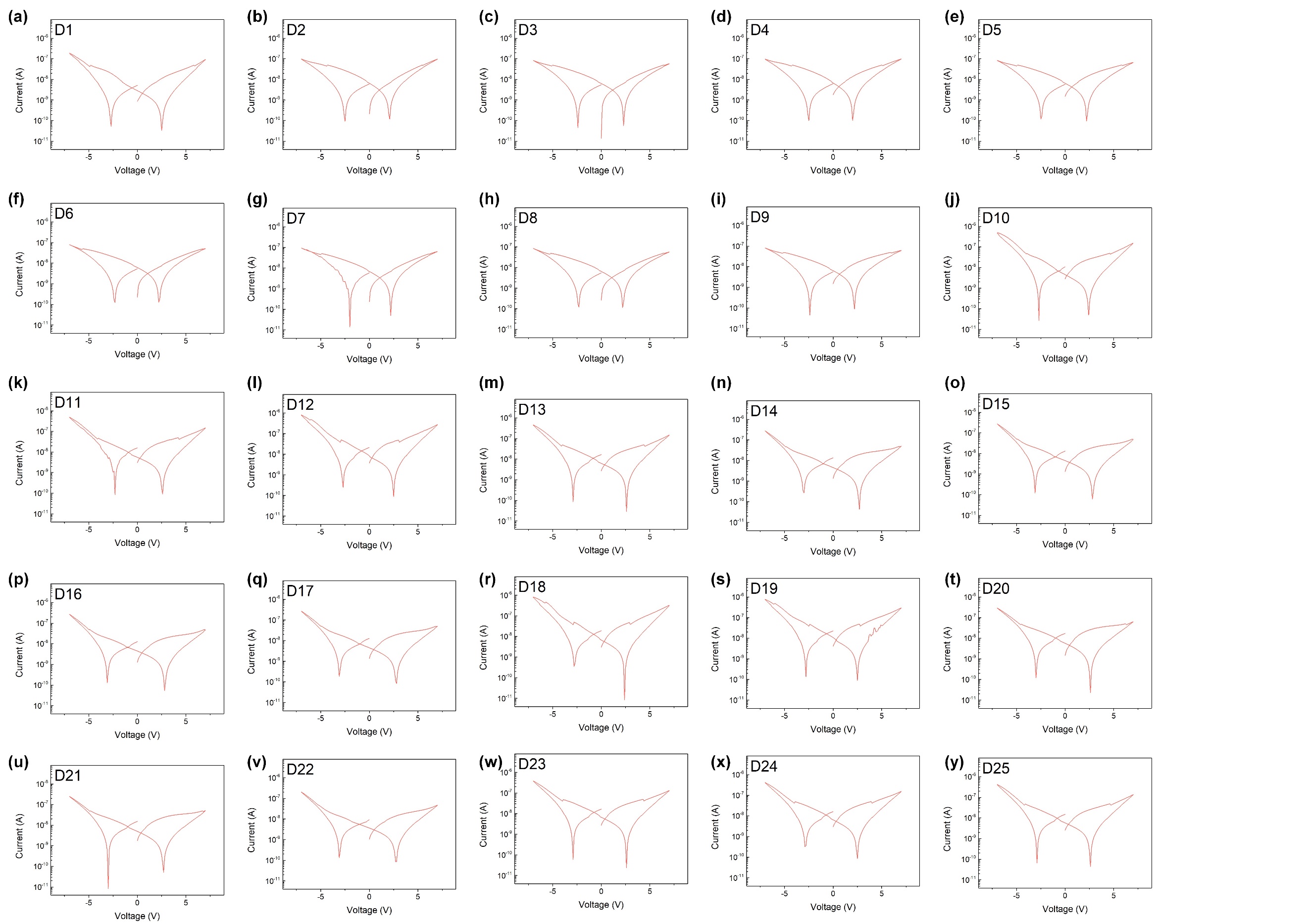


**Figure S6**. Device-to-device variability of 25 Hf-72 devices. Resistive characteristics of Devices 1 to 25, shown sequentially from (a) to (y).

**Figure S7**


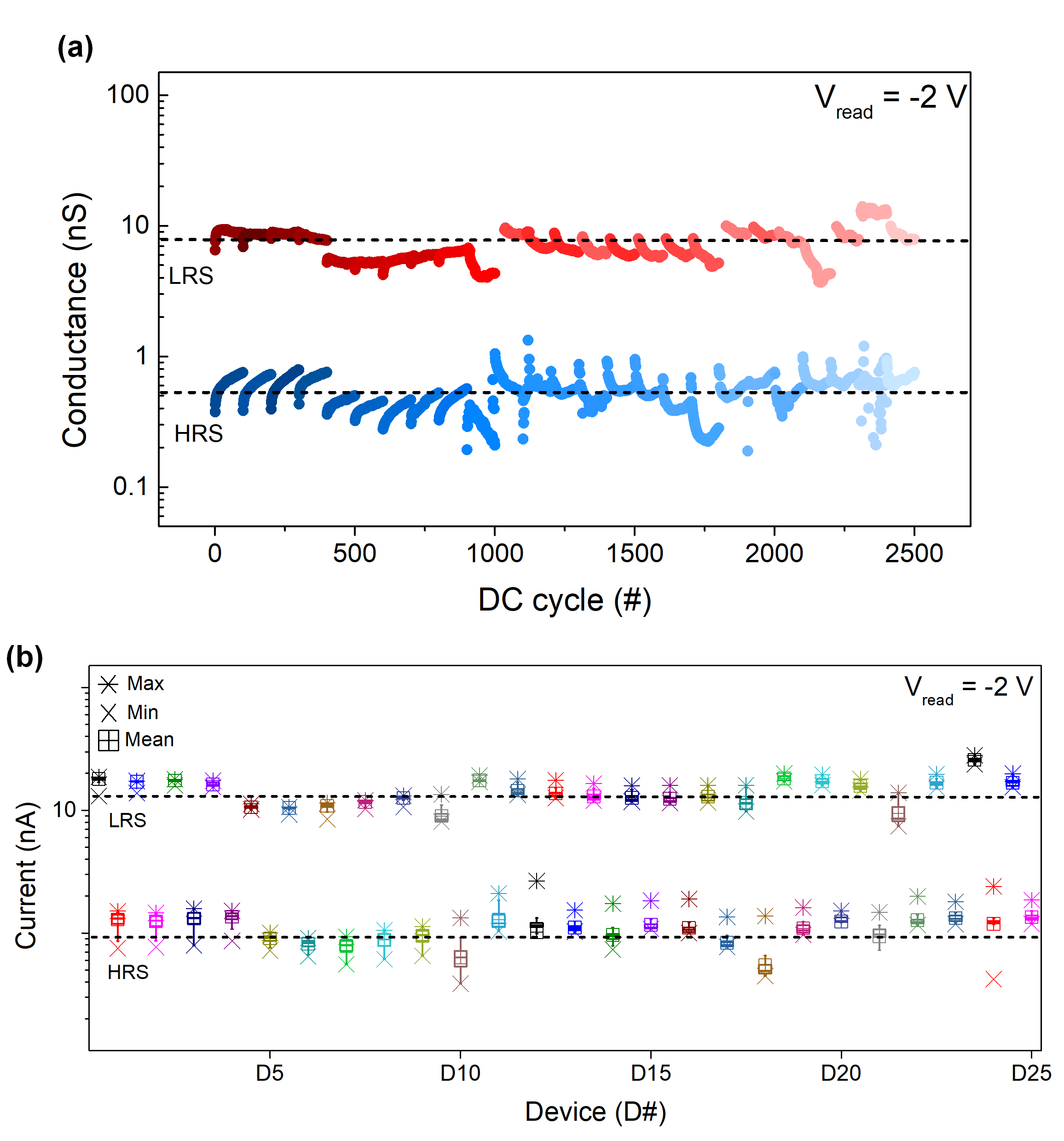


**Figure S7**. (a) Device-to-device uniformity analysis that shows 100 switching cycles for each of 25 different Hf-72 devices. (b) Distribution of the on-state current and off-state current for each of the 25 devices, each operated for 100 switching cycles

**Note S8**

First, the X-ray diffraction (XRD) analysis of the HfO_2_ active layers of each device was performed, which is shown in Figure S8a. The observed diffraction peaks matched well with the reference card for monoclinic HfO_2_ (JCPDS No. 06-0318) ^[17]^. No clear diffraction peaks were observed for the Hf-18 sample, which indicates a fully amorphous state. A prominent peak that corresponds to the (111) plane in contrast began to emerge around 28.2°for the Hf-45 and Hf-72 samples. The peak intensity in particular significantly increased for the Hf-72 device, which was in conjunction with the additional diffraction peaks that were observed at approximately 55.5°, 58.4°, and 62.2°. These findings suggest enhanced crystallinity due to increased atomic aggregation within the thin film, because the cumulative sputtering energy increases, which aligns well with the previous studies ^[15]^. However, the overall spectra remained predominantly amorphous, which suggest incomplete transition toward a polycrystalline phase. Nevertheless, as the kinetic energy from ion bombardment during sputtering converts into thermal energy and accumulates within the chamber, small grains or randomly oriented nuclei may form, which potentially leads to non-uniform polycrystallinity. The grain size (D) of each device was calculated using the Scherrer equation, which is provided below ^[18]^.

$$D = \frac{n\lambda}{\beta cos\theta}$$

where n is the Scherrer constant (0.90), λ is the X-ray wavelength (0.15406 nm), β is the full-width at half-maximum, and θ is the Bragg diffraction angle. The average grain size of the HfO_2_ thin films increased up to 0.31 nm, which is shown in the inset of Figure S8a.

Significant alterations in the surface morphology were expected considering the microstructural changes that were confirmed by the XRD analysis. The surface morphology of each HfO_2_ film was therefore visualized using atomic force microscopy (AFM). The AFM images that are presented in Figure S8b–d clearly demonstrate notable differences among the samples. The Hf-18 sample exhibited a relatively smooth and uniform surface with small grain sizes. The surface roughness (Ra) conversely progressively increased from 2.23 nm (Hf-18) to 3.21 nm (Hf-45) and further to 4.00 nm (Hf-72), which included corresponding irregular fluctuations in the grain height distributions ^[19]^. These results can be interpreted as morphological changes that resulted from increased crystallinity in the thin films, which is consistent with the XRD analysis.

Lastly, X-ray photoelectron spectroscopy (XPS) depth profiling was performed in order to examine the internal oxygen vacancy states within each HfO_2_ film by monitoring the O 1s spectra, which is shown in Figure S8e–g. The Ar⁺ ion beam energy was adjusted according to film thickness to control the depth rate in order to accurately analyze the core region of each HfO_2_ film, whereas the etching time was consistently maintained at one minute. This setup ensured the acquisition of signals that specifically target the central region of each film. The O 1s XPS spectra were deconvoluted into two components with binding energies at 530.4 eV and 531.2 eV, which were attributed to stoichiometric oxygen (M–O bonds) and oxygen vacancies, respectively. The oxygen vacancy ratios in the bulk of each sample were observed to gradually decrease, which included 46.2% for Hf-18, 31.8% for Hf-45, and 15.0% for Hf-72. These results indicate that the reduction in grain boundaries suppresses defect formation within the crystal lattice as the crystallinity improves, which thereby leads to a decrease in the oxygen vacancy concentration ^[20]^.

It was evident by integrating the XRD, AFM, and XPS analysis results that the crystallinity of the HfO_2_ active layers improved as the sample transitioned from Hf-18 to Hf-45 and further to Hf-72, whereas the internal oxygen vacancy concentration decreased. These observations collectively imply that variations in the electrical switching characteristics previously observed among the devices originate primarily from changes in the crystallinity and defect density within the HfO_2_ films.

**Figure S8**


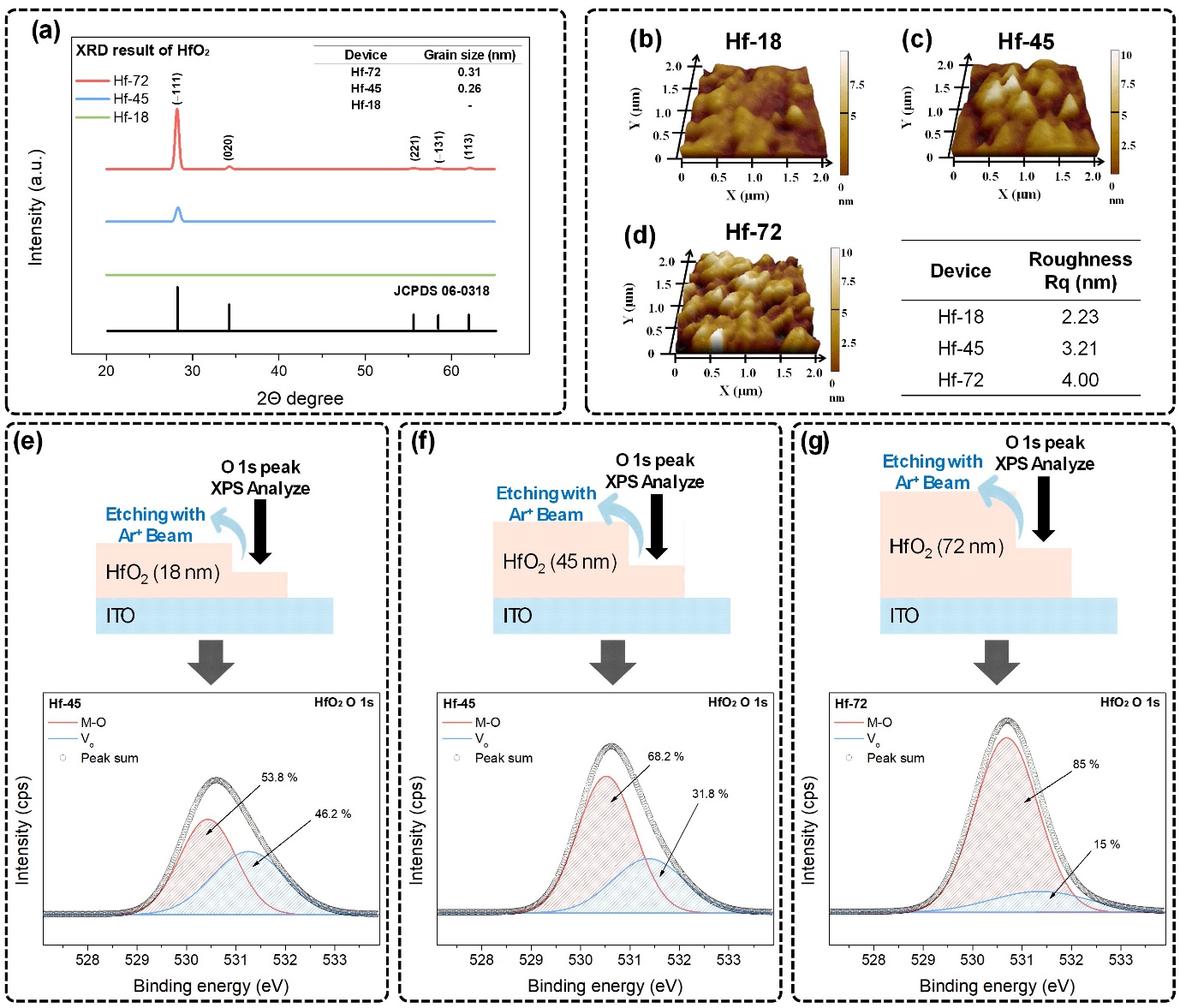


**Figure S8**. Material characteristic of Hf-18, Hf-45 and Hf-72. (a) XRD patterns of the HfO_2_ layers that correspond to the Hf-18, Hf-45, and Hf-72. AFM images of the HfO_2_ layers that correspond to the (b) Hf-18, (c) Hf-45, and (d) Hf-72. XPS O 1s spectra of the central regions of the HfO_2_ layers in (e) Hf-18, (f) Hf-45, and (g) Hf-72.

**Note S9**

The stepwise dynamic mechanism of the Hf-18 device, which operates based on the conventional filament-based RRAM model, is provided below.

Step 1: Formation (set) of a conductive filament is due to oxygen ion migration driven by electrical energy.

Step 2: The device transitions to a LRS as a result of the filament formation.

Step 3: Dissolution (reset) of the conductive filament occurs under an electric field of opposite polarity.

Step 4: The device transitions back to a HRS following the filament dissolution.

**Figure S9**


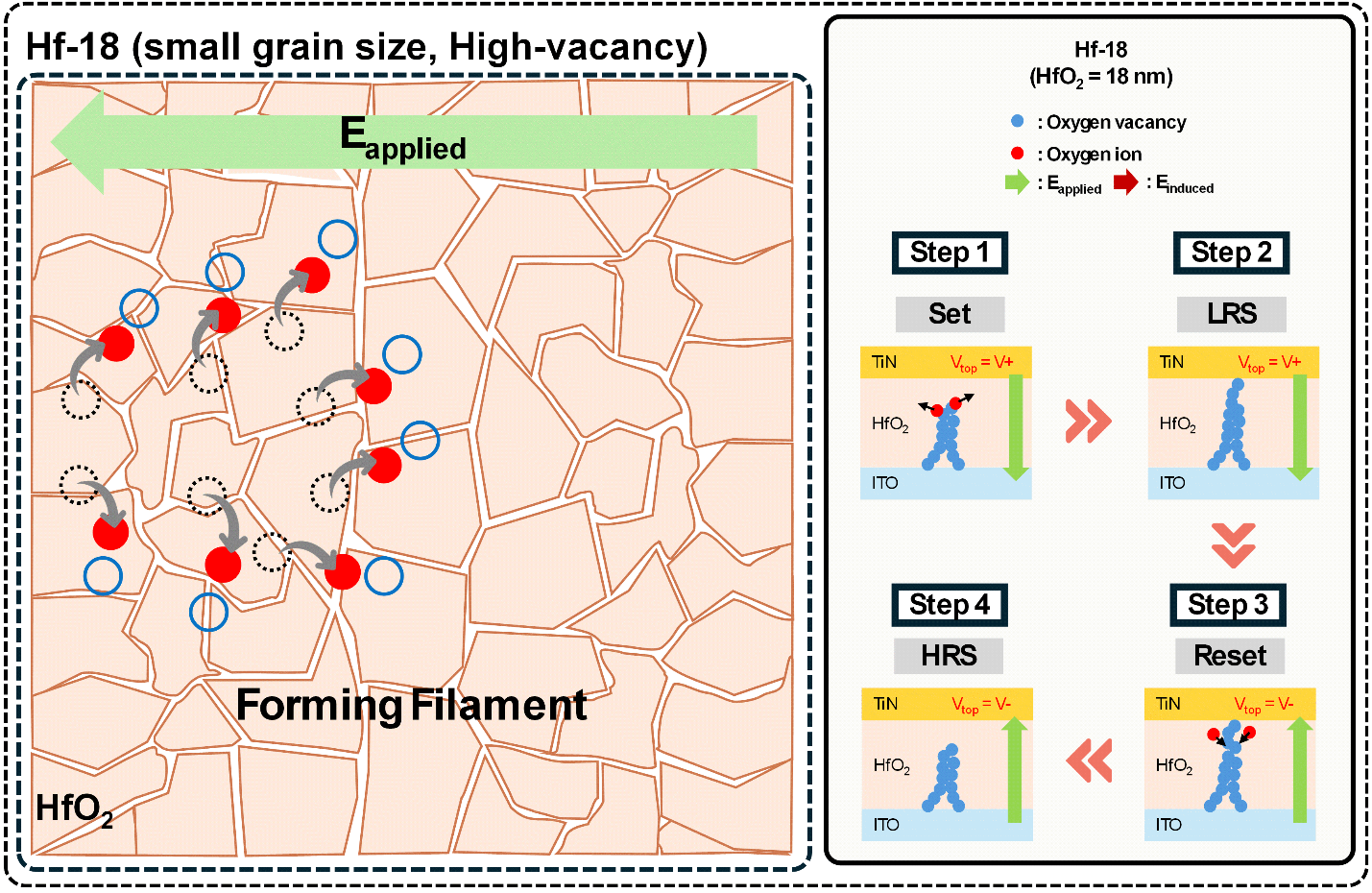


**Figure S9**. Schematic illustration of the switching mechanisms of the filamentary conduction in Hf-18.

**Figure S10**

***
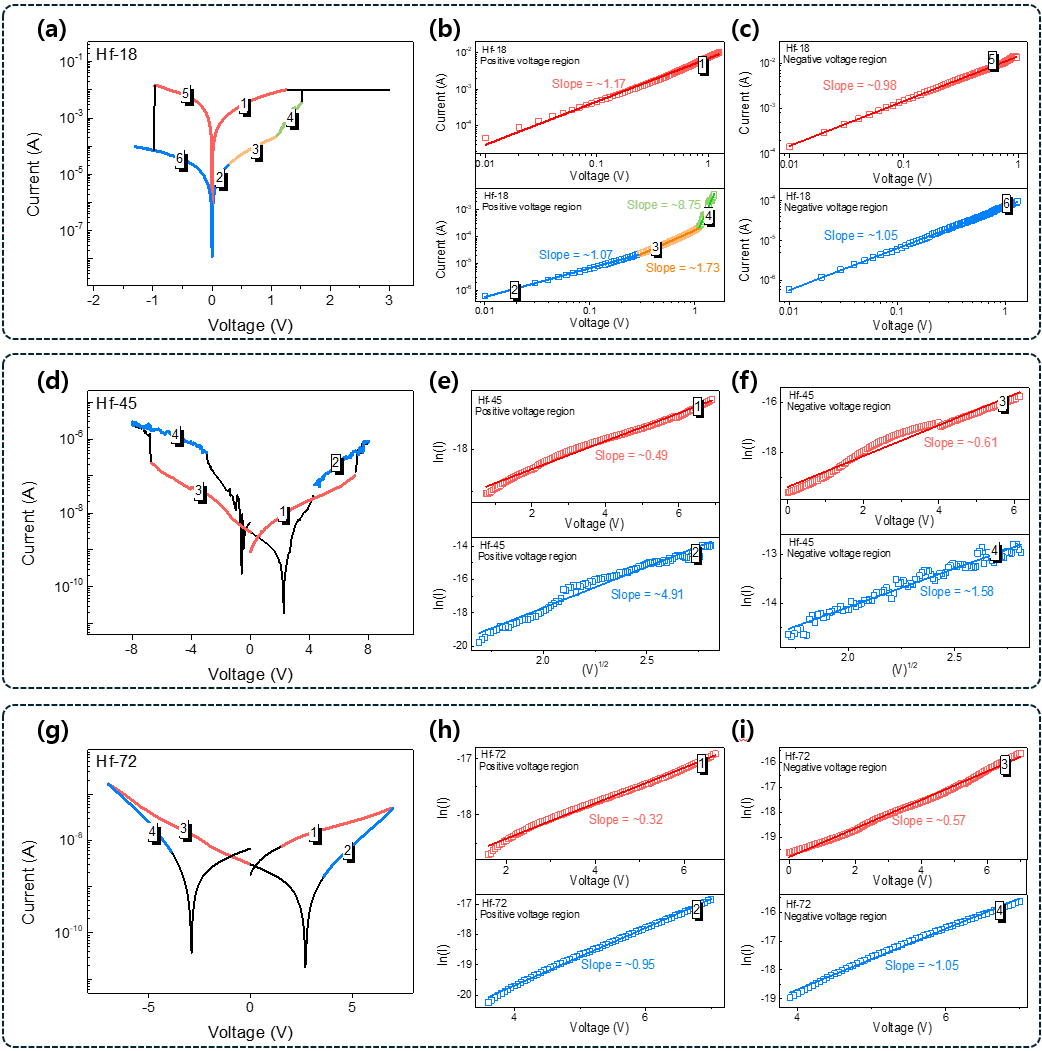
***

**Figure S10.** (a) *I–V* curves of Hf-18. (b) Typical *I–V* curves plotted by different fittings Hf-18 in (a) positive (SCLC) and (c) negative (Ohmic) branches. (c) *I–V* curves of Hf-45. (d) Typical *I–V* curves plotted by different fittings Hf-45 in (e) positive (electron tunneling, Schottky) and (f) negative (electron tunneling, Schottky) branches. (g) *I–V* curves of Hf-72. (d) Typical *I–V* curves plotted by different fittings Hf-72 in (e) positive (electron tunneling) and (f) negative (electron tunneling) branches.

**Note S11**

Figure S11 shows how the interaction between the induced electric field (𝐸_induced_) and the externally applied read field (𝐸_read_) changes depending on the read polarity after a positive DC voltage sweep. This diagram provides a physical explanation for the polarity-dependent bipolar bimodal switching behavior observed in the Hf-72 device.

**Left panel (Positive read after positive sweep):** After a positive DC sweep, applying a positive read voltage (+V_read_) creates a read field (𝐸_read_) that goes against the direction of the induced field (𝐸_induced_), which is generated by the dipoles that are aligned inside. The figure shows that these two fields partially cancel each other out, which lowers the total electric field (𝐸_total_). Because of this, the device has less current and appears to be in the HRS. The induced field slowly fades over time, which weakens the cancellation effect. This, in turn, increases conductivity, demonstrating a relaxation process.

**Right panel (Negative read after positive sweep):** When a negative read voltage (–V_read_) is applied to the same polarized condition, the read field (𝐸_read_) aligns with the induced field (𝐸_induced_), which makes the total field (𝐸_total_) stronger. This stronger field makes it easier for electrons to enter, and at first, the device looks like it's in an LRS. The induced field gets weaker over time, weakening the reinforcement effect and makes the device less conductive, which is a sign of relaxation.

This polarity-dependent field interaction mechanism reveals that even under the same initial dipole alignment, simply changing the read polarity can lead to distinct conductive states. This is the core operating principle behind the device’s bipolar bimodal switching behavior. Unlike conventional unidirectional volatile memory devices, this mechanism enables richer information extraction from a single physical state via multiple readouts, making it highly advantageous for neuromorphic and reservoir computing applications.

**Figure S11**


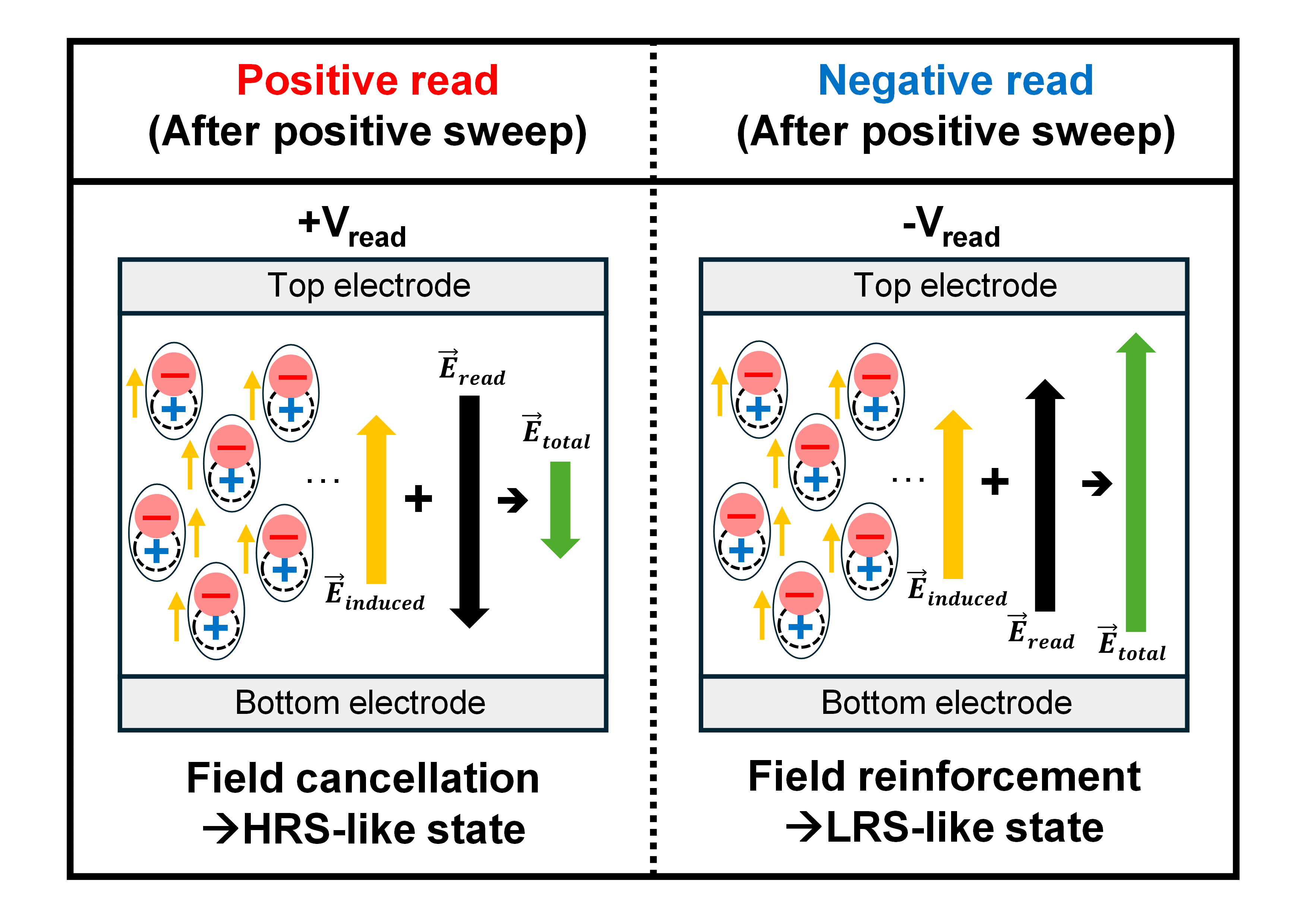


**Figure S11.** Conceptual illustration of dipole alignment under different read polarities after a positive sweep.

**Note S12**

We quantitatively analyzed how the field-induced dipole-based relaxation behavior of the Hf-72 device varies with temperature. For this purpose, an identical DC sweep voltage was applied and while measuring time-dependent conductance changes under the temperature conditions of 298, 323, 348, and 373 K. Figure S12a shows the relaxation behavior following a positive sweep (+7 V) and negative read voltage (–2 V), while Figure S12b corresponds to a negative sweep (–7 V) followed by a positive read voltage (+2 V).

At all temperatures, the device showed a typical relaxation trend, with the initial high conductance gradually decreasing over time. This behavior reflects the dissipation of field-induced dipoles, leading to a weakening of the internal electric field and a gradual return to the high-resistance state. The relaxation curves for each condition were well-fitted by a single exponential decay function, $y = A₁ exp(-x / \tau)$ ^[21]^ with R² values exceeding 0.96, indicating high fitting accuracy. Here, τ is the time constant of relaxation, which shows how long it takes for the system to get back to its initial state. The graphs in the inset show how τ changes with temperature in both figures. In all cases, τ increased as the temperature went up. This is attributed to oxygen ions (O²⁻) moving more easily at higher temperatures, which facilitates dipole alignments becoming stronger or spreading out more. ^[22]^. Notably, this trend was consistent across both positive and negative readout conditions, suggesting that the relaxation behavior is predominantly governed by field-induced dipole-related changes in the internal electric field.

**Figure S12**


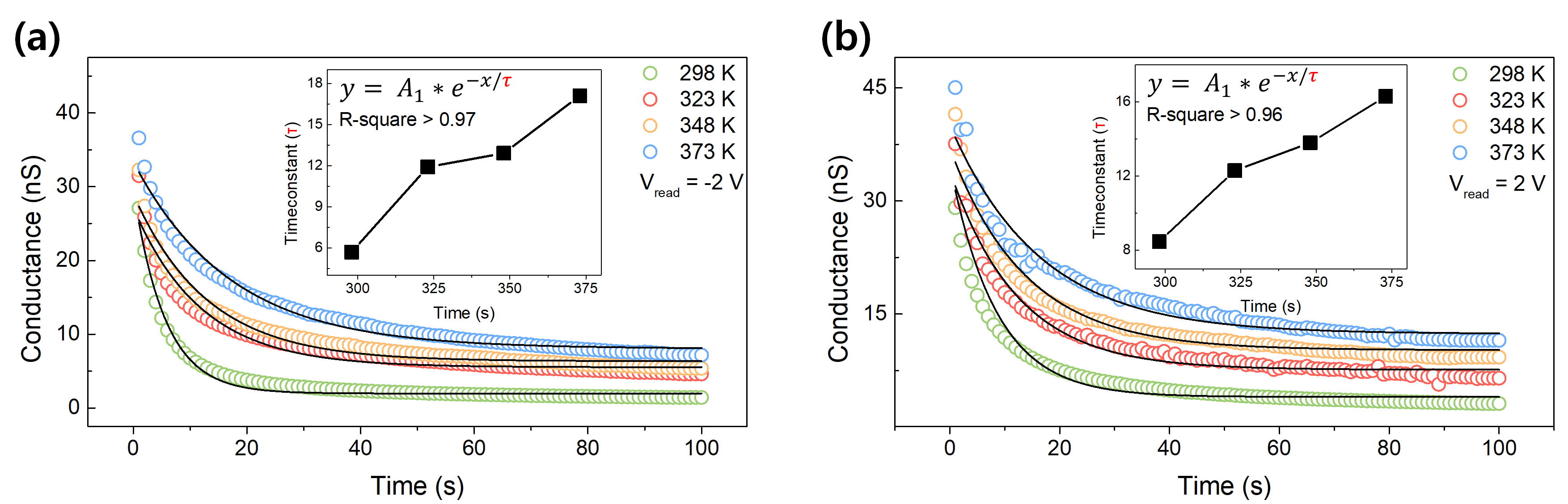


**Figure S12.** Temperature-dependent conductance relaxation of the Hf-72 device under (a) −2 V (after 7 V sweep) and (b) +2 V read voltages (after −7 V sweep). Insets show extracted time constants (τ), increasing with temperature, fitted by single exponential decay.

**Figure S13**


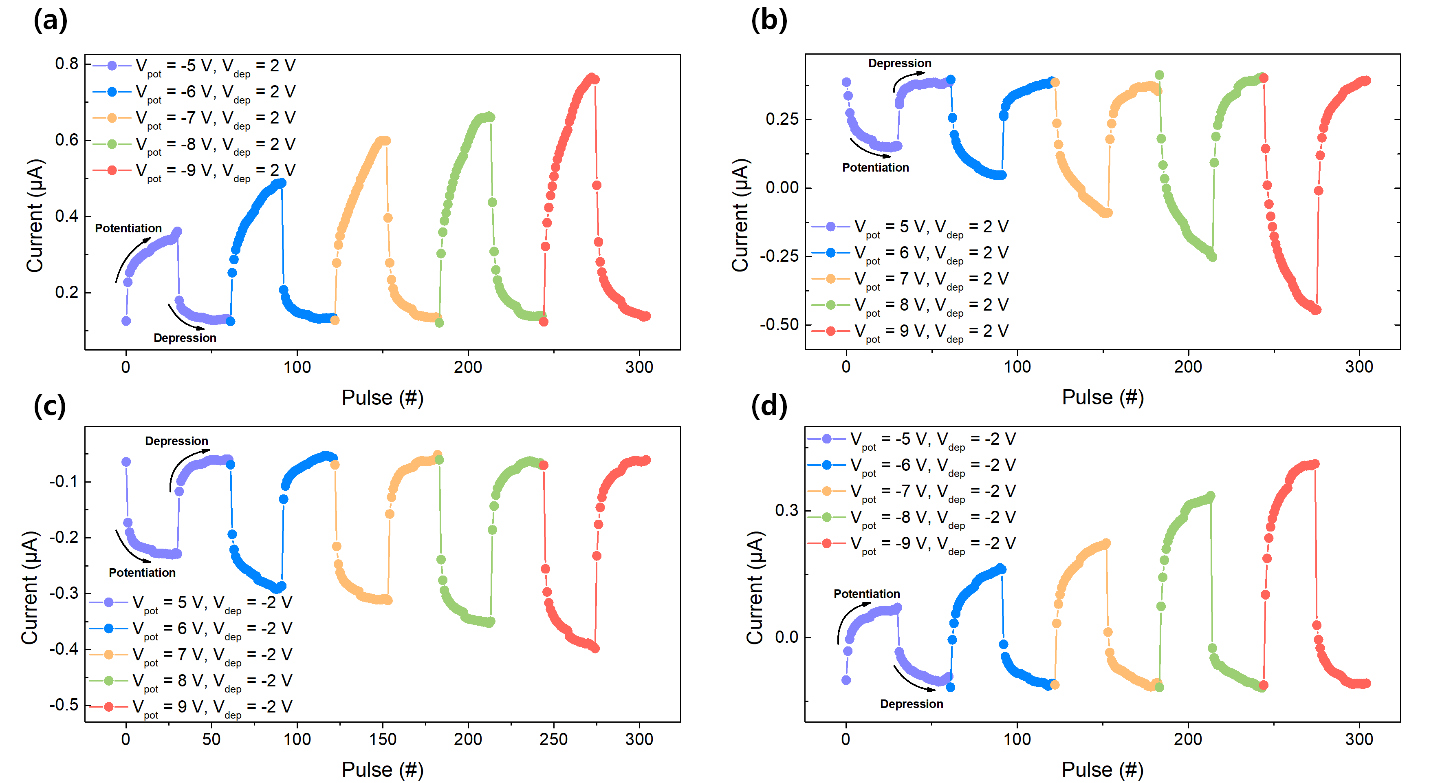


**Figure S13.** Potentiation and depression curves:(a) V_pot_ = –5 ~ –9 V , V_dep_ = 2 V (b) V_pot_ = 5 ~ 9 V , V_dep_ = 2 V, (c) V_pot_ = 5 ~ 9 V , V_dep_ = –2 V, (d) V_pot_ = –5 ~ –9 V , V_dep_ = –2 V.

**Note S14**

In this study, the proposed reservoir computing system was evaluated using both positive and negative write pulses. Although the Hf-72 device exhibits a highly symmetric I–V characteristic, the actual power consumption for processing a single input is more appropriately calculated as the average of the power consumed under both polarities. The input voltage was set to ±7 V, and the corresponding currents were –0.32 μA (for –7 V) and 0.31 μA (for 7 V), respectively. These current values were extracted from the midpoint of each input pulse (I_middle_), as shown in Figure S13. Based on this, the average power consumption is estimated as (–7 V × –0.32 μA + 7 V × 0.31 μA) ÷ 2 ≈ 2.205 μW ^[23]^. Given the pulse width of 0.8 ms used in the system, the energy consumption per input pulse is calculated as approximately 1.764 nJ. These results suggest that further improvements in energy efficiency are possible by optimizing the input voltage amplitude or pulse duration in future implementations. Moreover, recent studies published in leading journals over the past five years were reviewed, and compiled representative cases were compiled in Table S2, where the energy consumption per input was calculated using the same method. Comparative analysis indicates that our device demonstrates competitive performance in terms of both energy efficiency and temporal dynamics, highlighting its potential for practical and efficient reservoir computing applications.

**Figure S14**


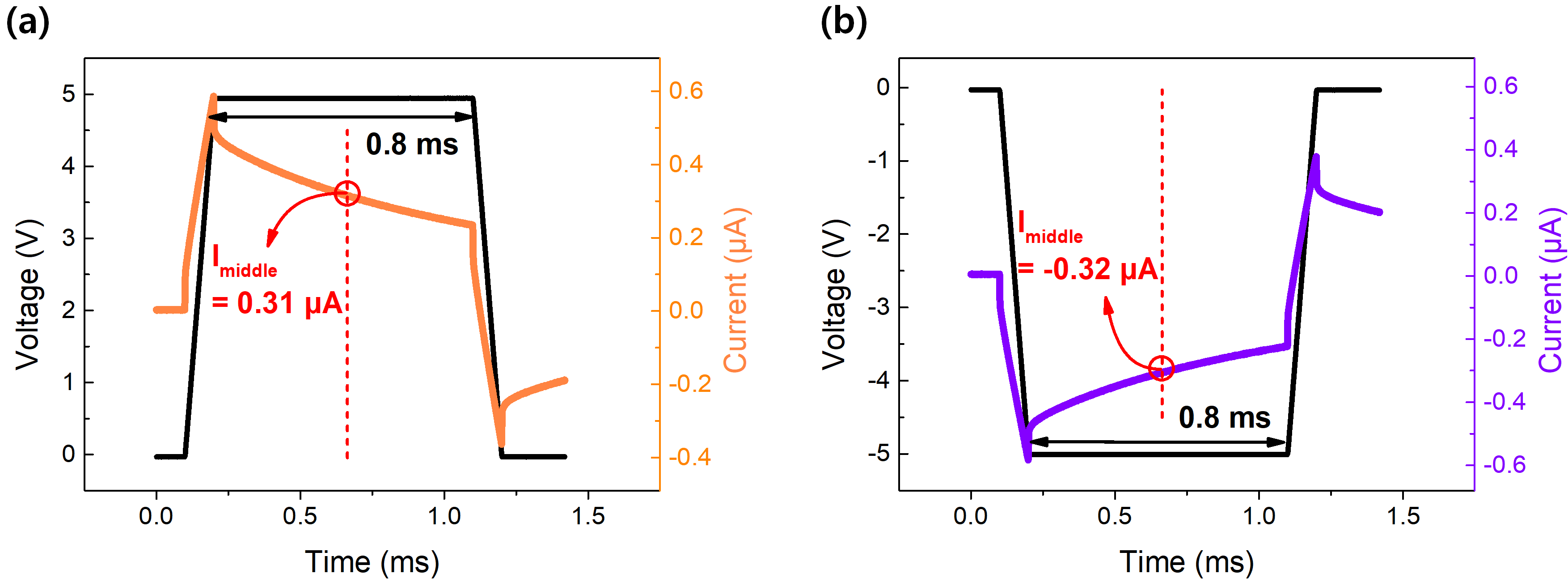


**Figure S14**. Measurement of voltage and current during pulse application for energy estimation.
The current measured at the midpoint of the 0.8 ms pulse was used to calculate power and energy consumption under (a) positive and (b) negative bias conditions.

**Table S2**

| **No.** | **Structure** | **Power per input** | **Energy per input** | **Pulse width** | **Ref** |
| --- | --- | --- | --- | --- | --- |
| 1 | ITO/ZrO_x_/TaN | ~ 6.4 μW | ~32 nJ | 5 ms | ^[3]^ |
| 2 | Ti/TiO_x_/TaO_y_/Pt | ~ 50 μW | ~ 6 nJ | 0.12 ms | ^[23]^ |
| 3 | Au/Cr/SnS/CrAu/SiO_2_/Si | 990 μW | 19.8 μJ | 20 ms | ^[24]^ |
| 4 | Ta/Ti/TaO_x_/Pt | ~ 27.5 mW | 27.5 μJ | 1 ms | ^[25]^ |
| 5 | TiN/HfO_2_/Pt | ~ 15 μW | 1.5 μJ | 100 ms | ^[26]^ |
| 6 | STO/SRO/BFO/Pt | ~ 11.8 μW | ~ 23.6 nJ | 2 ms | ^[27]^ |
| 7 | Ti/NbO_x_/Pt/SiO_2_/Si | ~ 33 μW | 33 nJ | 1 ms | ^[28]^ |
| 8 | Ti/TiO_x_/Pd | ~ 19.2 μW | ~ 42.24 nJ | 2.2 ms | ^[29]^ |
| 9 | **TiN/HfO_2_/ITO** | **~ 2.205** μW | **~ 1.764 nJ** | **0.8ms** | **This work** |

**Table S2**. Summary of power consumption and energy per input operation for recent memristor-based neuromorphic devices

**Figure S15**


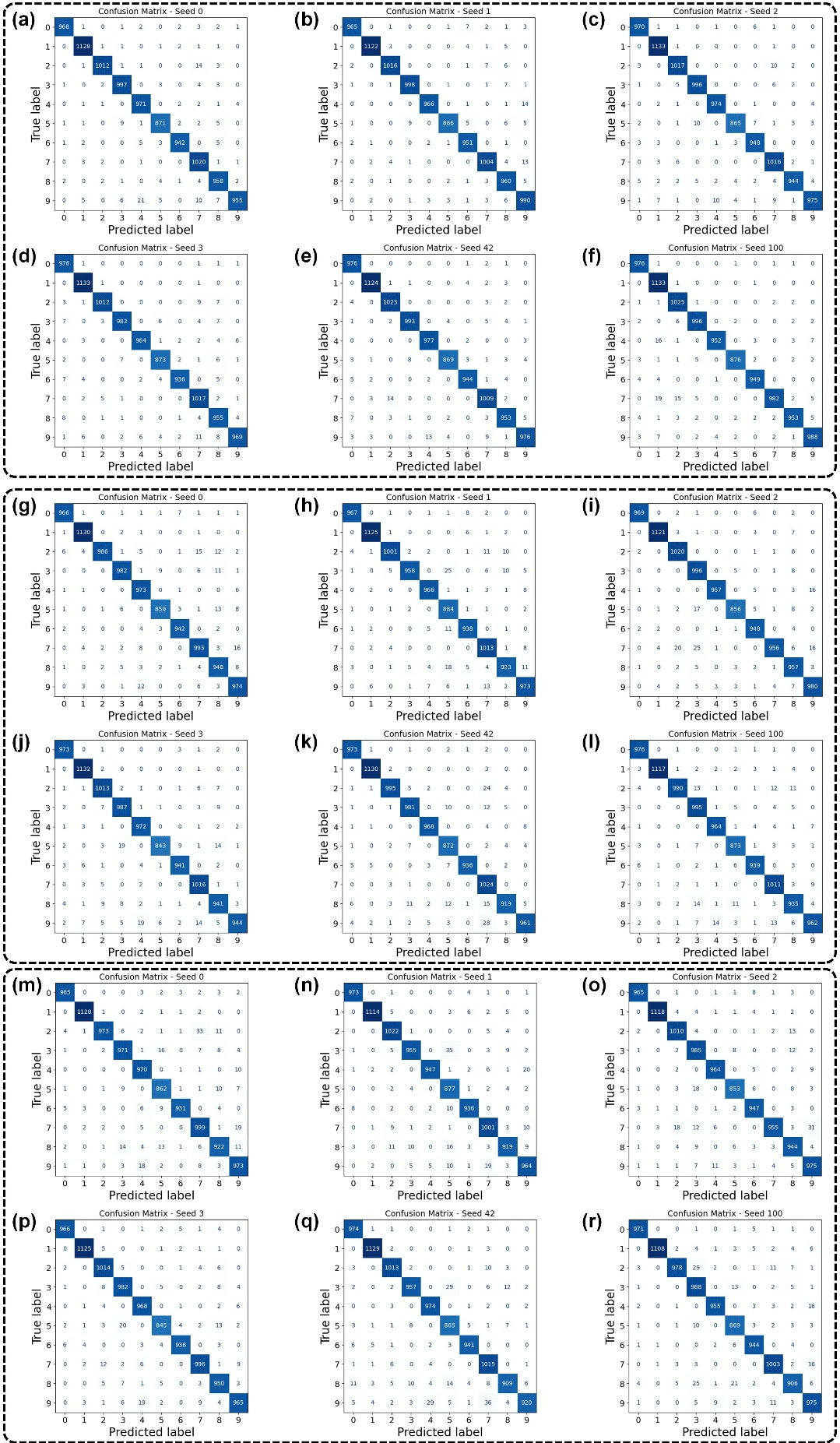


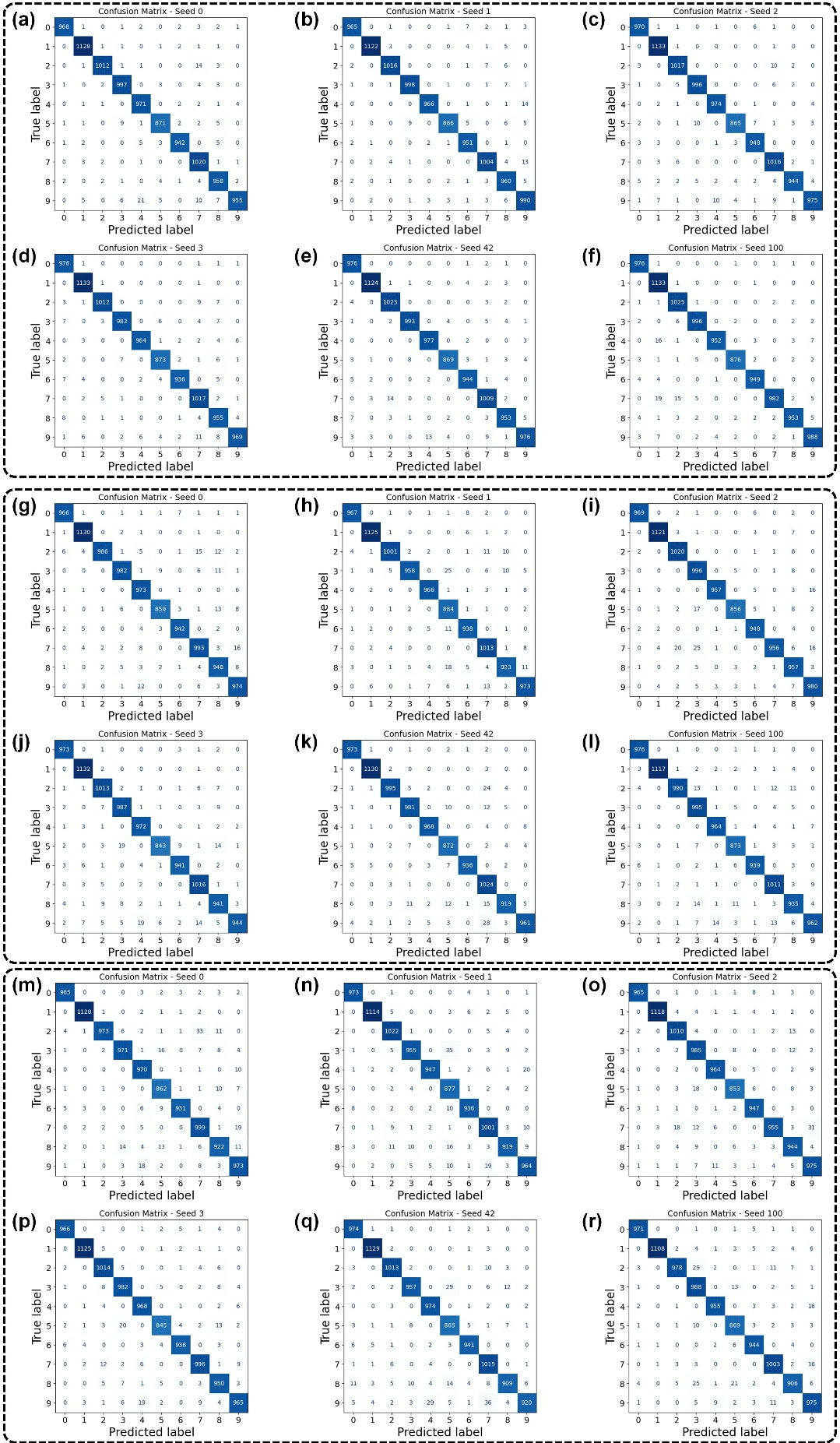


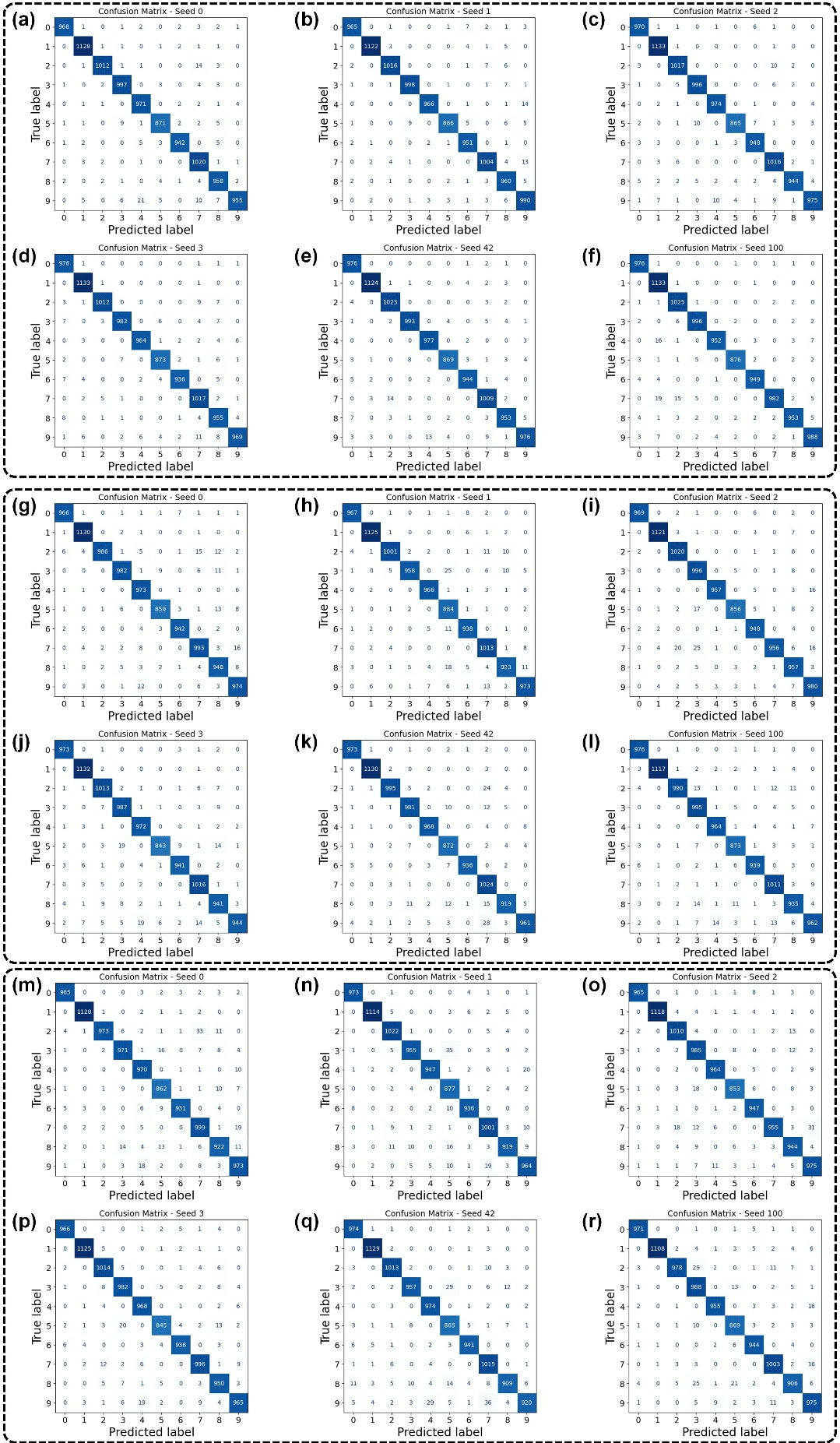


**Figure S15**. Confusion matrices of the RC-based pattern classification results when defining a binary 1 using positive polarity input pulses. (a)–(f) Three-channel input using +G, –G, and G′, (g)–(l) single-channel input using +G only, and (m)–(r) single-channel input using –G only.

**Figure S16**


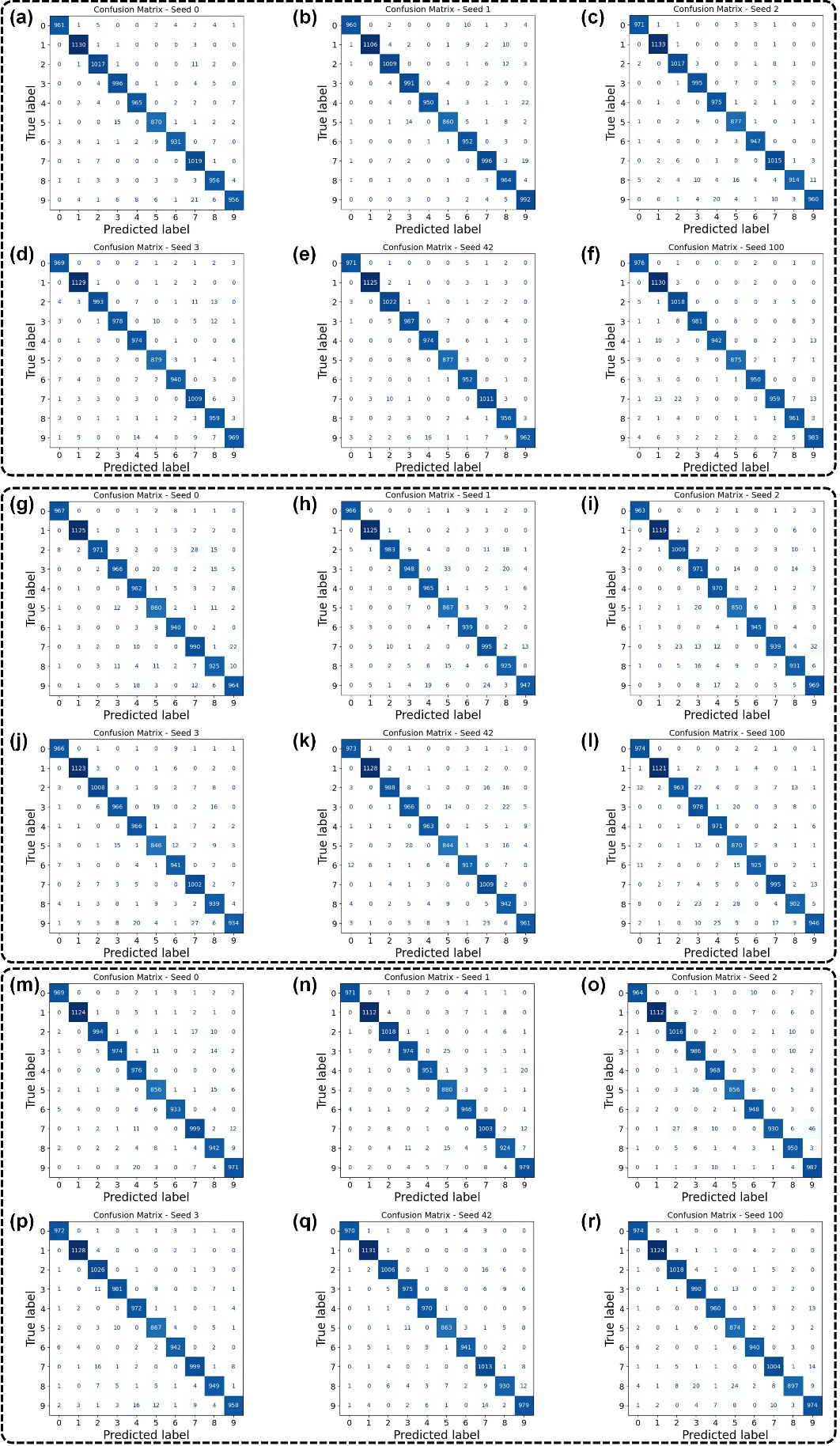


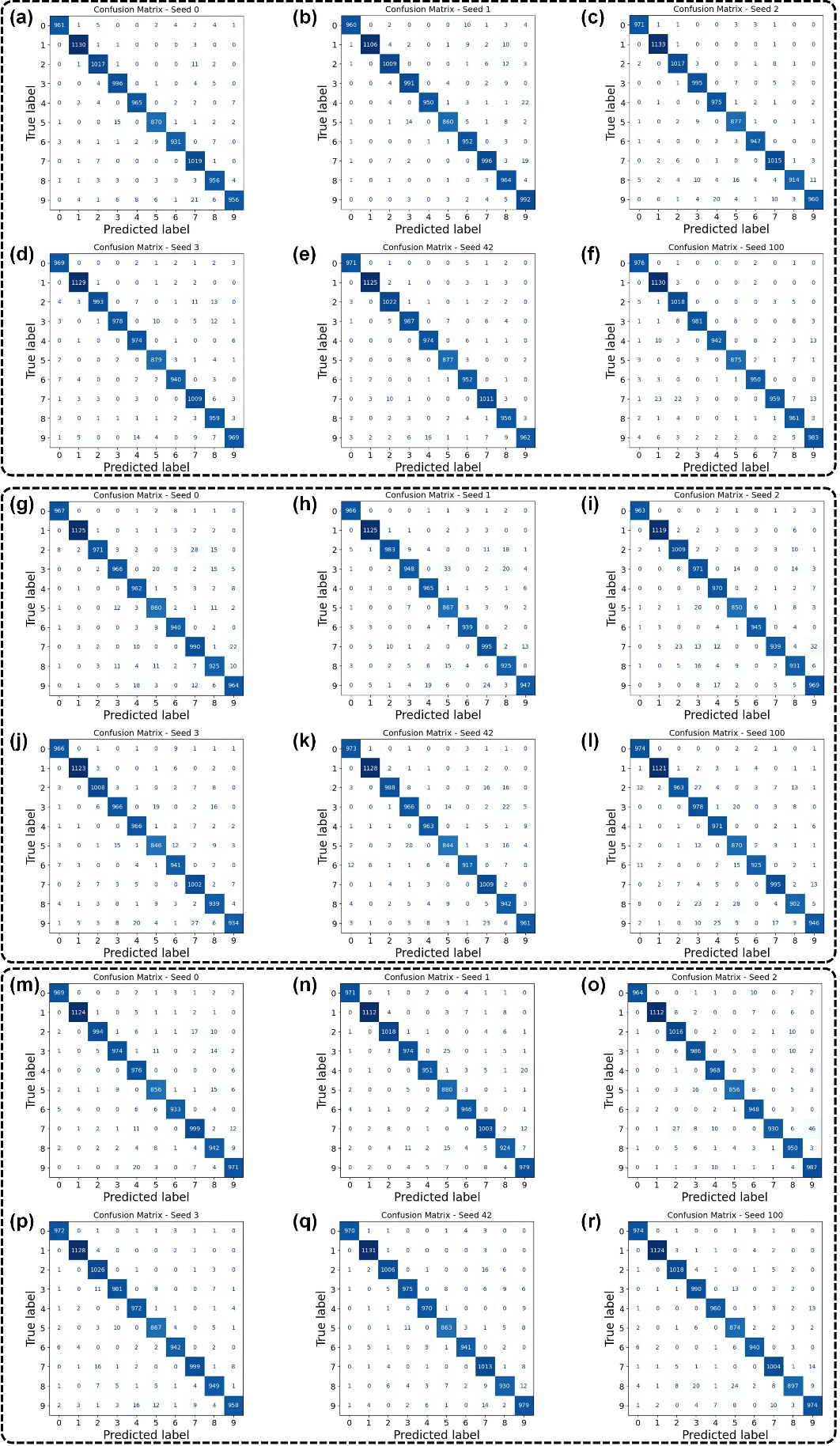


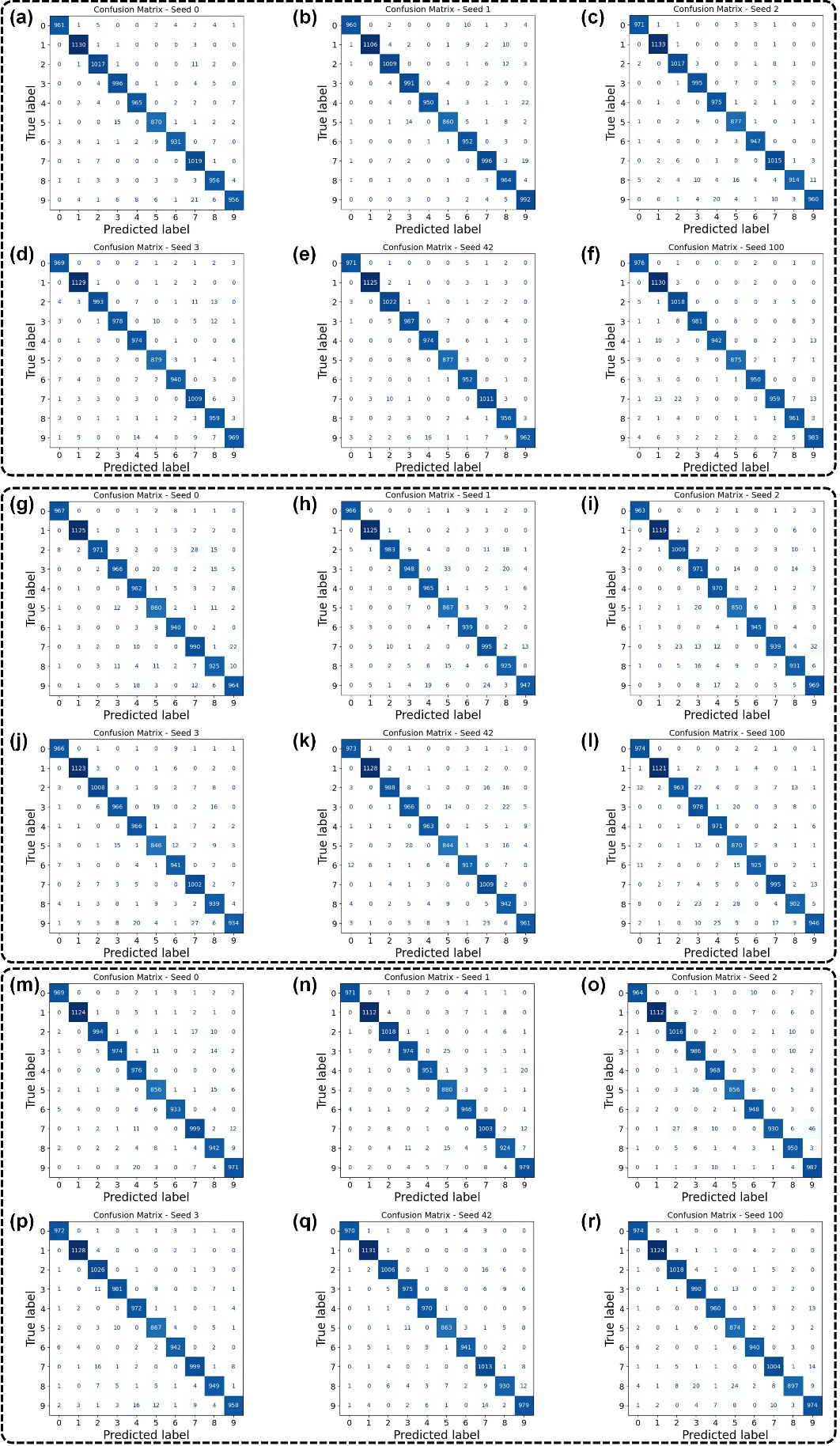


**Figure S16**. Confusion matrices of the RC-based pattern classification results when defining a binary 1 using negative polarity input pulses. (a)–(f) Three-channel case using (+G, –G, G′), (g)–(l) single-channel case using only +G, and (m)–(r) single-channel case using only –G.

**Figure S17**


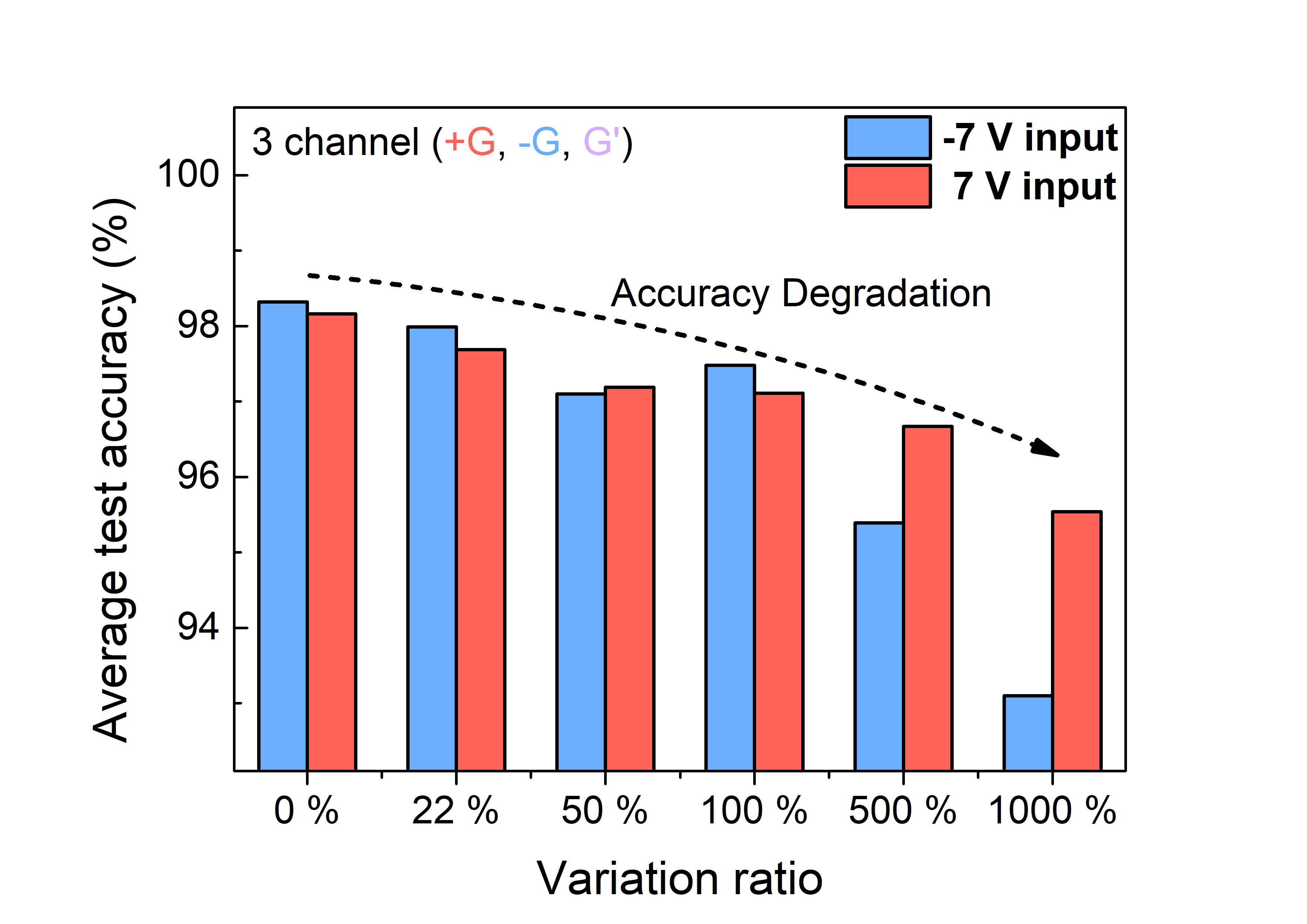


**Figure S17.** Effect of increasing structural heterogeneity on classification performance in the 3-channel reservoir (+G, –G, G′).

**Figure S18**


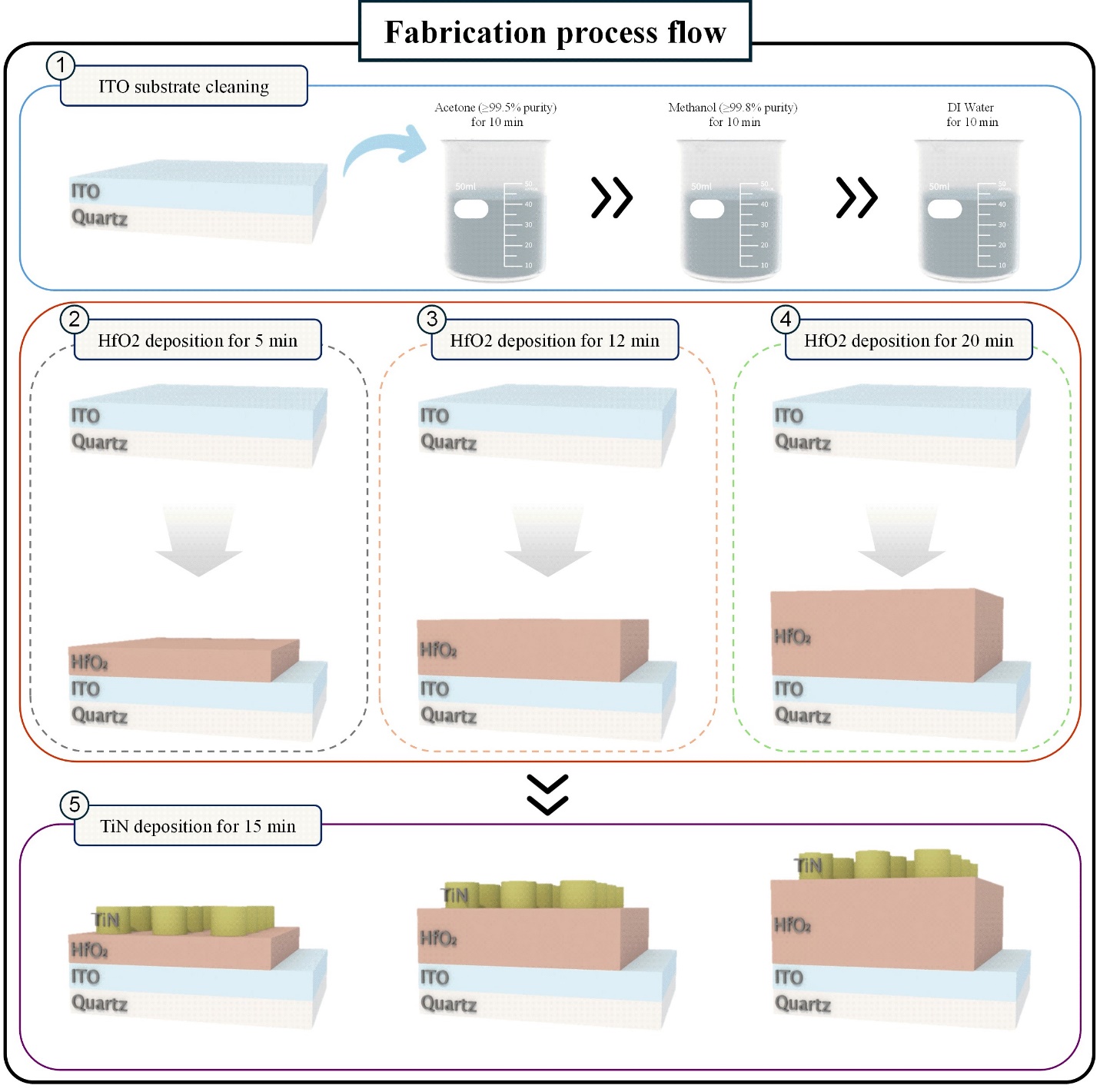


**Figure S18**. Fabrication process flow for TiN/HfO_2_/ITO memristor devices with varying sputtering times.

**Figure S19**


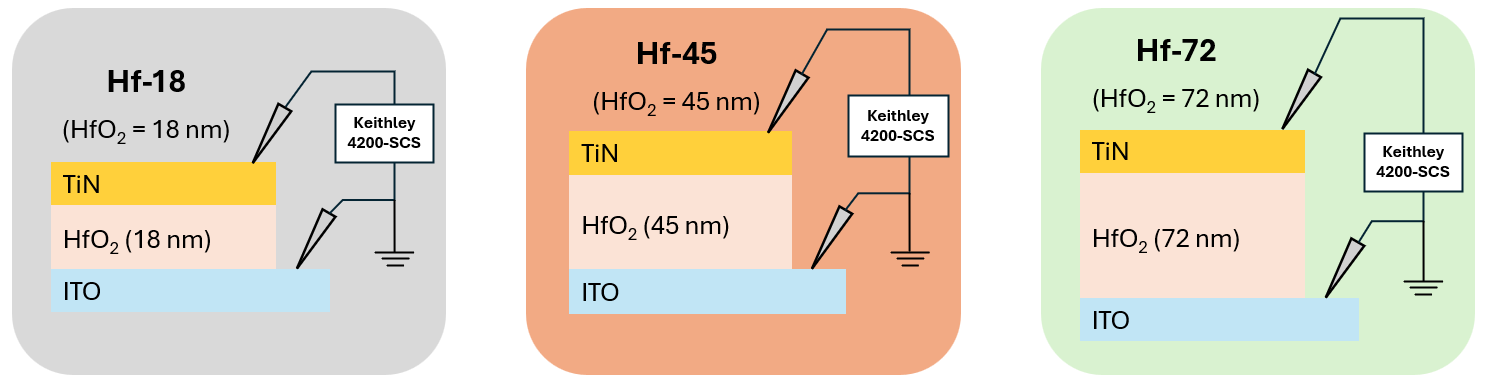


**Figure S19**. Schematic illustrations of the electrical measurement setup for HfO_2_-based devices.

**SI Reference**

[1] D. Ju, M. Noh, S. Lee, G. Kim, J. Park, S. Kim. “Self‐Rectifying Volatile Memristor for Highly Dynamic Functions.” Advanced Functional Materials, no. (2025): 2423880.

[2] N. Ghenzi, T. W. Park, S. S. Kim, H. J. Kim, Y. H. Jang, K. S. Woo, C. S. Hwang. “Heterogeneous reservoir computing in second-order Ta 2 O 5/HfO 2 memristors.” Nanoscale Horizons 9, no. 3 (2024): 427.

[3] D. Ju, M. Koo, S. Kim. “Implementation of 8-bit reservoir computing through volatile ZrOx-based memristor as a physical reservoir.” Nano Energy 128, no. (2024): 109958.

[4] W. Hu, Z. Fan, L. Mo, H. Lin, M. Li, W. Li, J. Ou, R. Tao, G. Tian, M. Qin. “Volatile Resistive Switching and Short-Term Synaptic Plasticity in a Ferroelectric-Modulated SrFeO x Memristor.” ACS Applied Materials & Interfaces, no. (2025).

[5] C. Du, F. Cai, M. A. Zidan, W. Ma, S. H. Lee, W. D. Lu. “Reservoir computing using dynamic memristors for temporal information processing.” Nature communications 8, no. 1 (2017): 2204.

[6] M. Noh, H. Park, S. Kim. “Dynamic resistive switching of WOx-based memristor for associative learning activities, on-receptor, and reservoir computing.” Chaos, Solitons & Fractals 196, no. (2025): 116381.

[7] H. Jang, D. Ju, S. Kim. “Demonstration of cognitive learning, associative learning, and multi-bit reservoir computing using TiOx/HfOx-based volatile memristor with low current.” Journal of Alloys and Compounds, no. (2025): 178897.

[8] B. Cai, T. Wang, C. Wang, Q. Sun, D. W. Zhang, L. Chen. “A Photomemristor With Temporal Dynamics for In-Sensor Reservoir Computing.” IEEE Electron Device Letters 45, no. 4 (2024): 570.

[9] D. Sharma, A. Luqman, S. E. Ng, N. Yantara, X. Xing, Y. B. Tay, A. Basu, A. Chattopadhyay, N. Mathews. “Halide perovskite photovoltaics for in-sensor reservoir computing.” Nano Energy 129, no. (2024): 109949.

[10] J. Song, J. Meng, C. Lu, T. Wang, C. Wan, H. Zhu, Q. Sun, D. W. Zhang, L. Chen. “Self-powered optoelectronic synaptic device for both static and dynamic reservoir computing.” Nano Energy 134, no. (2025): 110574.

[11] D. K. Lee, G. Noh, S. Oh, Y. Jo, E. Park, M. J. Kim, D. Y. Woo, H. Wi, Y. Jeong, H. J. Jang. “Crystallinity‐controlled volatility tuning of ZrO2 memristor for physical reservoir computing.” InfoMat 7, no. 2 (2025): e12635.

[12] M. Ismail, S. Lee, M. Rasheed, C. Mahata, S. Kim. “Exploitation of temporal dynamics and synaptic plasticity in multilayered ITO/ZnO/IGZO/ZnO/ITO memristor for energy-efficient reservoir computing.” Journal of Materials Science & Technology 235, no. (2025): 37.

[13] D. Ju, J. Lee, H. So, S. Kim. “TiN/TiOx/WOx/Pt heterojunction memristor for sensory and neuromorphic computing.” Journal of Alloys and Compounds 1004, no. (2024): 175830.

[14] R. Mandal, A. Mandal, T. Som. “Towards on-receptor computing: Electronic nociceptor embedded neuromorphic functionalities at nanoscale.” Applied Materials Today 37, no. (2024): 102103.

[15] J. Hu, J. Wang, Y. Wei, Q. Wu, F. Zhang, Q. Xu. “Effect of film growth thickness on the refractive index and crystallization of HfO2 film.” Ceramics International 47, no. 23 (2021): 33751.

[16] Q. N. Chen, Y. Ou, F. Ma, J. Li. “Mechanisms of electromechanical coupling in strain based scanning probe microscopy.” Applied Physics Letters 104, no. 24 (2014).

[17] A. Ramadoss, S. J. Kim. “Synthesis and characterization of HfO2 nanoparticles by sonochemical approach.” Journal of alloys and compounds 544, no. (2012): 115.

[18] B. D. Cullity, R. Smoluchowski. “Elements of X‐ray Diffraction.” Physics Today 10, no. 3 (1957): 50.

[19] J. W. Park, P. S. Jeong, S.-H. Choi, H. Lee, B. H. Kong, H. K. Cho. “Optical and structural properties of ion-implanted InGaZnO thin films studied with spectroscopic ellipsometry and transmission electron microscopy.” Japanese Journal of Applied Physics 48, no. 11R (2009): 111603.

[20] Y. Wang, K.-M. Kang, M. Kim, H.-H. Park. “Film thickness effect in c-axis oxygen vacancy-passivated ZnO prepared via atomic layer deposition by using H2O2.” Applied Surface Science 529, no. (2020): 147095.

[21] R. Hu, X. Li, J. Tang, Y. Li, X. Zheng, B. Gao, H. Qian, H. Wu. “Investigation of Resistive Switching Mechanisms in Ti/TiOx/Pd‐Based RRAM Devices.” Advanced Electronic Materials 8, no. 8 (2022): 2100827.

[22] G. Wang, C. Li, Y. Chen, Y. Xia, D. Wu, Q. Xu. “Reversible voltage dependent transition of abnormal and normal bipolar resistive switching.” Scientific reports 6, no. 1 (2016): 36953.

[23] Y. Zhong, J. Tang, X. Li, B. Gao, H. Qian, H. Wu. “Dynamic memristor-based reservoir computing for high-efficiency temporal signal processing.” Nature communications 12, no. 1 (2021): 408.

[24] L. Sun, Z. Wang, J. Jiang, Y. Kim, B. Joo, S. Zheng, S. Lee, W. J. Yu, B.-S. Kong, H. Yang. “In-sensor reservoir computing for language learning via two-dimensional memristors.” Science advances 7, no. 20 (2021): eabg1455.

[25] G. Milano, G. Pedretti, K. Montano, S. Ricci, S. Hashemkhani, L. Boarino, D. Ielmini, C. Ricciardi. “In materia reservoir computing with a fully memristive architecture based on self-organizing nanowire networks.” Nature materials 21, no. 2 (2022): 195.

[26] Y. H. Jang, S. H. Lee, J. Han, W. Kim, S. K. Shim, S. Cheong, K. S. Woo, J. K. Han, C. S. Hwang. “Spatiotemporal data processing with memristor crossbar‐array‐based graph reservoir.” Advanced Materials 36, no. 7 (2024): 2309314.

[27] Z. Chen, W. Li, Z. Fan, S. Dong, Y. Chen, M. Qin, M. Zeng, X. Lu, G. Zhou, X. Gao. “All-ferroelectric implementation of reservoir computing.” Nature communications 14, no. 1 (2023): 3585.

[28] D. Ju, S. Kim. “Versatile NbOx‐Based Volatile Memristor for Artificial Intelligent Applications.” Advanced Functional Materials 34, no. 49 (2024): 2409436.

[29] Y. Zhong, J. Tang, X. Li, X. Liang, Z. Liu, Y. Li, Y. Xi, P. Yao, Z. Hao, B. Gao. “A memristor-based analogue reservoir computing system for real-time and power-efficient signal processing.” Nature Electronics 5, no. 10 (2022): 672.
